# The spread of low-credibility content by social bots

**DOI:** 10.1038/s41467-018-06930-7

**Published:** 2018-11-20

**Authors:** Chengcheng Shao, Giovanni Luca Ciampaglia, Onur Varol, Kai-Cheng Yang, Alessandro Flammini, Filippo Menczer

**Affiliations:** 1 School of Informatics, Computing, and Engineering, Indiana University Bloomington, Bloomington, 47408 IN USA; 2 College of Computer, National University of Defense Technology, Changsha, 410073 Hunan China; 3 Indiana University Network Science Institute, Bloomington, 47408 IN USA

## Abstract

The massive spread of digital misinformation has been identified as a major threat to democracies. Communication, cognitive, social, and computer scientists are studying the complex causes for the viral diffusion of misinformation, while online platforms are beginning to deploy countermeasures. Little systematic, data-based evidence has been published to guide these efforts. Here we analyze 14 million messages spreading 400 thousand articles on Twitter during ten months in 2016 and 2017. We find evidence that social bots played a disproportionate role in spreading articles from low-credibility sources. Bots amplify such content in the early spreading moments, before an article goes viral. They also target users with many followers through replies and mentions. Humans are vulnerable to this manipulation, resharing content posted by bots. Successful low-credibility sources are heavily supported by social bots. These results suggest that curbing social bots may be an effective strategy for mitigating the spread of online misinformation.

## Abstract (web summary)

Online misinformation is a threat to a well-informed electorate and undermines democracy. Here, the authors analyse the spread of articles on Twitter, find that bots play a major role in the spread of low-credibility content and suggest control measures for limiting the spread of misinformation.

## Introduction

As we access news from social media^1^, we are exposed to a daily dose of false or misleading news reports, hoaxes, conspiracy theories, click-bait headlines, junk science, and even satire^2^. We refer to such content collectively as “misinformation.” The financial incentives through advertising are well understood^3^, but political motives can be equally powerful^4,5^. The massive spread of digital misinformation has been identified as a major global risk^6^ and alleged to influence elections and threaten democracies^7^. While such claims are hard to prove^8^, real harm of disinformation has been demonstrated in health and finance^9,10^.

Social and computer scientists are engaged in efforts to study the complex mix of cognitive, social, and algorithmic biases that make us vulnerable to manipulation by online misinformation^11^. These include information overload and finite attention^12^, novelty of false news^2^, the selective exposure^13–15^ caused by polarized and segregated online social networks^16,17^, algorithmic popularity bias^18–20^, and other cognitive vulnerabilities such as confirmation bias and motivated reasoning^21–23^.

Abuse of online information ecosystems can both exploit and reinforce these vulnerabilities. While fabricated news are not a new phenomenon^24^, the ease with which social media can be manipulated^5^ creates novel challenges and particularly fertile grounds for sowing disinformation^25^. Public opinion can be influenced thanks to the low cost of producing fraudulent websites and high volumes of software-controlled profiles, known as social bots^10,26^. These fake accounts can post content and interact with each other and with legitimate users via social connections, just like real people^27^. Bots can tailor misinformation and target those who are most likely to believe it, taking advantage of our tendencies to attend to what appears popular, to trust information in a social setting^28^, and to trust social contacts^29^. Since earliest manifestations uncovered in 2010^4,5^, we have seen influential bots affect online debates about vaccination policies^10^ and participate actively in political campaigns, both in the United States^30^ and other countries^31,32^.

The fight against online misinformation requires a grounded assessment of the relative impact of different mechanisms by which it spreads. If the problem is mainly driven by cognitive limitations, we need to invest in news literacy education; if social media platforms are fostering the creation of echo chambers, algorithms can be tweaked to broaden exposure to diverse views; and if malicious bots are responsible for many of the falsehoods, we can focus attention on detecting this kind of abuse. Here we focus on gauging the latter effect. With few exception^2,30,32,33^, the literature about the role played by social bots in the spread of misinformation is largely based on anecdotal or limited evidence; a quantitative understanding of the effectiveness of misinformation-spreading attacks based on social bots is still missing. A large-scale, systematic analysis of the spread of misinformation by social bots is now feasible thanks to two tools developed in our lab: the Hoaxy platform to track the online spread of claims^33^ and the Botometer machine learning algorithm to detect social bots^26^. Here we examine social bots and how they promote the spread of misinformation through millions of Twitter posts during and following the 2016 US presidential campaign. We find that social bots amplify the spread of misinformation by exposing humans to this content and inducing them to share it.

## Results

### Low-credibility content

Our analysis is based on a large corpus of news stories posted on Twitter. Operationally, rather than focusing on individual stories that have been debunked by fact-checkers, we consider low-credibility content, i.e., content from low-credibility sources. Such sources are websites that have been identified by reputable third-party news and fact-checking organizations as routinely publishing various types of low-credibility information (see Methods). There are two reasons for this approach^11^. First, these sources have processes for the publication of disinformation: they mimic news media outlets without adhering to the professional standards of journalistic integrity. Second, fact-checking millions of individual articles is unfeasible. As a result, this approach is widely adopted in the literature (see Supplementary Discussion).

We track the complete production of 120 low-credibility sources by crawling their websites and extracting all public tweets with links to their stories. Our own analysis of a sample of these articles confirms that the vast majority of low-credibility content is some type of misinformation (see Methods). We also crawled and tracked the articles published by seven independent fact-checking organizations. The present analysis focuses on the period from mid-May 2016 to the end of March 2017. During this time, we collected 389,569 articles from low-credibility sources and 15,053 articles from fact-checking sources. We further collected from Twitter all of the public posts linking to these articles: 13,617,425 tweets linked to low-credibility sources and 1,133,674 linked to fact-checking sources. See Methods and Supplementary Methods for details.

### Spreading patterns and actors

**Fig. 1:**
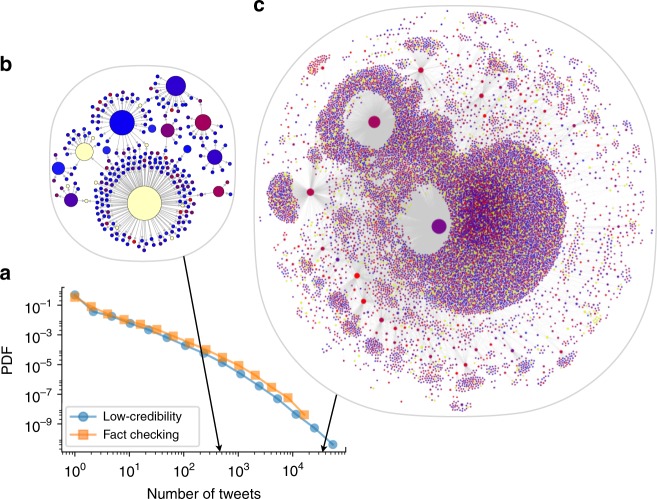
Online virality of content. **a** Probability distribution (density function) of the number of tweets for articles from both low-credibility (blue circles) and fact-checking (orange squares) sources. The distributions of the number of accounts sharing an article are very similar (see Supplementary Fig. 2). As illustrations, the diffusion networks of two stories are shown: **b** a medium-virality misleading article titled “FBI just released the Anthony Weiner warrant, and it proves they stole election”, published a month after the 2016 US election and shared in over 400 tweets; and **c** a highly viral fabricated news report titled “Spirit cooking”: Clinton campaign chairman practices bizarre occult ritual, published 4 days before the 2016 US election and shared in over 30,000 tweets. In both cases, only the largest connected component of the network is shown. Nodes and links represent Twitter accounts and retweets of the article, respectively. Node size indicates account influence, measured by the number of times an account was retweeted. Node color represents bot score, from blue (likely human) to red (likely bot); yellow nodes cannot be evaluated because they have either been suspended or deleted all their tweets. An interactive version of the larger network is available online (iunetsci.github.io/HoaxyBots/). Note that Twitter does not provide data to reconstruct a retweet tree; all retweets point to the original tweet. The retweet networks shown here combine multiple cascades (each a “star network” originating from a different tweet) that all share the same article link

On average, a low-credibility source published approximately 100 articles per week. By the end of the study period, the mean popularity of those articles was approximately 30 tweets per article per week (see Supplementary Fig. 1). However, as shown in Fig. 1, success is extremely heterogeneous across articles. Whether we measure success by number of posts containing a link (Fig. 1a) or by number of accounts sharing an article (Supplementary Fig. 2), we find a very broad distribution of popularity spanning several orders of magnitude: while the majority of articles goes unnoticed, a significant fraction goes “viral.” We observe that the popularity distribution of low-credibility articles is almost indistinguishable from that of fact-checking articles, meaning that low-credibility content is equally or more likely to spread virally. This result is similar to that of an analysis based on only fact-checked claims, which found false news to be even more viral than real news^2^. The qualitative conclusion is the same: links to low-credibility content reach massive exposure.

**Fig. 2:**
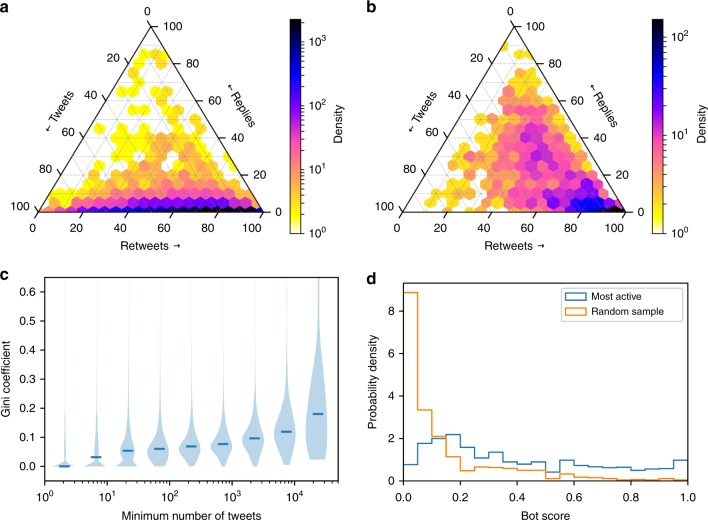
Anomalies. The distribution of types of tweet spreading articles from **a** low-credibility and **b** fact-checking sources are quite different. Each article is mapped along three axes representing the percentages of different types of messages that share it: original tweets, retweets, and replies. When user Alice retweets a tweet by user Bob, the tweet is rebroadcast to all of Alice’s followers, whereas when she replies to Bob’s tweet, the reply is only seen by Bob and users who follow them both. Color represents the number of articles in each bin, on a log scale. **c** Correlation between popularity of articles from low-credibility sources and concentration of posting activity. We consider a collection of articles shared by a minimum number of tweets as a popularity group. For articles in each popularity group, a violin plot shows the distribution of Gini coefficients which measure concentration of posts by few accounts (see Supplementary Methods). In violin plots, the width of a contour represents the probability of the corresponding value, and the median is marked by a colored line. **d** Bot score distributions for a random sample of 915 accounts who posted at least one link to a low-credibility source (orange), and for the 961 “super-spreaders” that most actively shared content from low-credibility sources (blue). The two groups have significantly different scores (*p* < 10^−4^ according to the Mann–Whitney *U* test): super-spreaders are more likely bots

Even though low-credibility and fact-checking sources show similar popularity distributions, we observe some distinctive patterns in the spread of low-credibility content. First, most articles by low-credibility sources spread through original tweets and retweets, while few are shared in replies (Fig. 2a); this is different from articles by fact-checking sources, which are shared mainly via retweets but also replies (Fig. 2b). In other words, the spreading patterns of low-credibility content are less “conversational.” Second, the more a story was tweeted, the more the tweets were concentrated in the hands of few accounts, who act as “super-spreaders” (Fig. 2c). This goes against the intuition that, as a story reaches a broader audience organically, the contribution of any individual account or group of accounts should matter less. In fact, a single account can post the same low-credibility article hundreds or even thousands of times (see Supplementary Fig. 6). This could suggest that the spread is amplified through automated means.

We hypothesize that the “super-spreaders” of low-credibility content are social bots which are automatically posting links to articles, retweeting other accounts, or performing more sophisticated autonomous tasks, like following and replying to other users. To test this hypothesis, we used Botometer to evaluate the Twitter accounts that posted links to articles from low-credibility sources. For each account we computed a bot score (a number in the unit interval) which can be interpreted as the level of automation of that account. We used a threshold of 0.5 to classify an account as bot or human. Details about the Botometer system and the threshold can be found in Methods. We first considered a random sample of the general population of accounts that shared at least one link to a low-credibility article. Only 6% of accounts in the sample are labeled as bots using this method, but they are responsible for spreading 31% of all tweets linking to low-credibility content, and 34% of all articles from low-credibility sources (Supplementary Table 2). We then compared this group with a sample of the top most active accounts (“super-spreaders”), 33% of which have been labeled as bot—over five times as many (details in Supplementary Methods). Figure 2d confirms that the super-spreaders are significantly more likely to be bots compared to the general population of accounts who share low-credibility content. Because these results are based on a classification model, it is important to make sure that what we see in Fig. 2d is not due to bias in the way Botometer was trained—that the model did not simply learn to assign higher scores to more active accounts. We rule out this competing explanation by showing that higher bot scores cannot be attributed to this kind of bias in the learning model (see Supplementary Fig. 16).

### Bot strategies

**Fig. 3:**
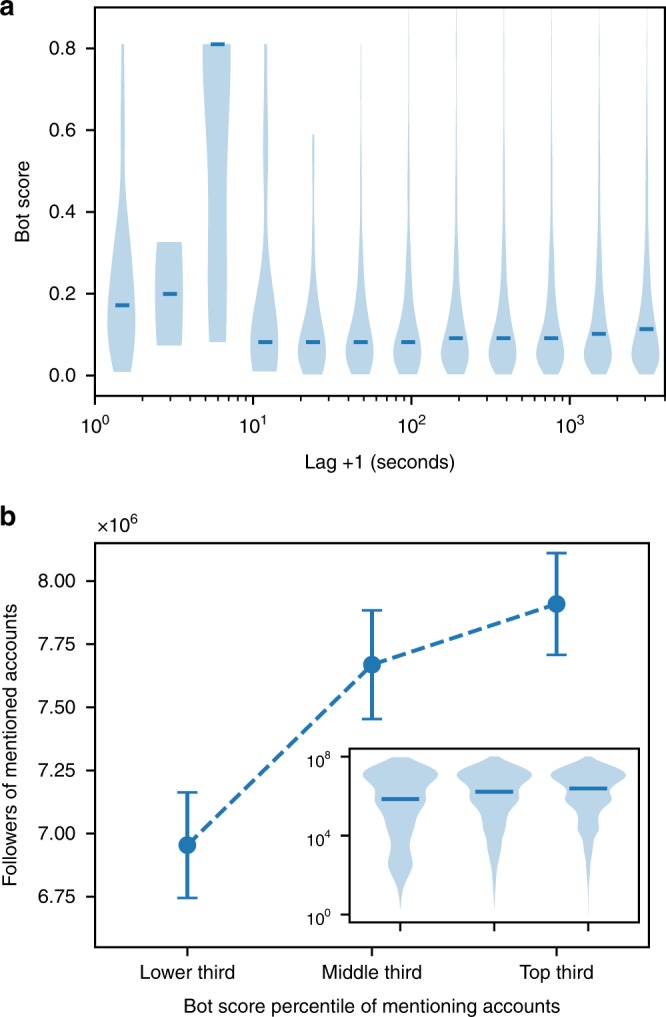
Bot strategies. **a** Early bot support after a viral low-credibility article is first shared. We consider a sample of 60,000 accounts that participate in the spread of the 1000 most viral stories from low-credibility sources. We align the times when each article first appears. We focus on the 1 h early spreading phase following each of these events, and divide it into logarithmic lag intervals. The plot shows the bot score distribution for accounts sharing the articles during each of these lag intervals. **b** Targeting of influentials. We plot the average number of followers of Twitter users who are mentioned (or replied to) by accounts that link to the most viral 1000 stories. The mentioning accounts are aggregated into three groups by bot score percentile. Error bars indicate standard errors. Inset: Distributions of follower counts for users mentioned by accounts in each percentile group

Given this evidence, we submit that bots may play a critical role in driving the viral spread of content from low-credibility sources. To test this question, we examined whether bots tend to get involved at particular times in the spread of popular articles. As shown in Fig. 3a, likely bots are more prevalent in the first few seconds after an article is first published on Twitter than at later times. We conjecture that this early intervention exposes many users to low-credibility articles, increasing the chances than an article goes “viral.”

We find that another strategy often used by bots is to mention influential users in tweets that link to low-credibility content. Bots seem to employ this targeting strategy repetitively; for example, a single account mentioned @realDonaldTrump in 19 tweets, each linking to the same false claim about millions of votes by illegal immigrants (see details in Supplementary Discussion and Supplementary Fig. 7). For a systematic investigation, let us consider all tweets that mention or reply to a user and include a link to a viral article from a low-credibility source in our corpus. The number of followers is often used as a proxy for the influence of a Twitter user. As shown in Fig. 3b, in general tweets tend to mention popular people. However, accounts with the largest bot scores tend to mention users with a larger number of followers (median and average). A possible explanation for this strategy is that bots (or rather, their operators) target influential users with content from low-credibility sources, creating the appearance that it is widely shared. The hope is that these targets will then reshare the content to their followers, thus boosting its credibility.

### Bot impact

**Fig. 4:**
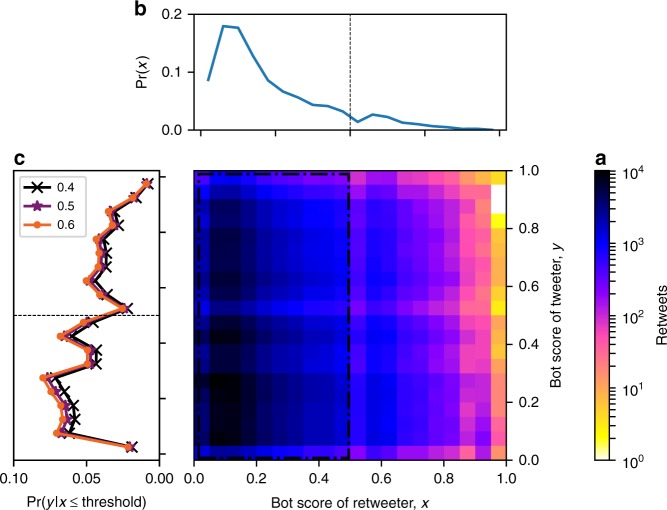
Impact of bots on humans. **a** Joint distribution of bot scores of accounts that retweeted links to low-credibility articles and accounts that had originally posted the links. Color represents the number of retweeted messages in each bin, on a log scale. **b** The top projection shows the distribution of bot scores for retweeters, who are mostly human. **c** The left projection shows the distribution of bot scores for accounts retweeted by likely humans who are identified by scores below a threshold of 0.4 (black crosses), 0.5 (purple stars), or 0.6 (orange circles). Irrespective of the threshold, we observe a significant portion of likely bots retweeted by likely humans

Having found that automated accounts are employed in ways that appear to drive the viral spread of low-credibility articles, let us explore how humans interact with the content shared by bots, which may provide insight into whether and how bots are able to affect public opinion. Figure 4a shows who retweets whom: humans do most of the retweeting (Fig. 4b), and they retweet articles posted by likely bots almost as much as those by other humans (Fig. 4c). This result, which is robust to the choice of threshold used to identify likely humans, suggests that collectively, people do not discriminate between low-credibility content shared by humans versus social bots. It also means that when we observe many accounts exposed to low-credibility information, these are not just bots (re)tweeting it. In fact, we find that the volume of tweets by likely humans scales super-linearly with the volume by likely bots, suggesting that the reach of these articles among humans is amplified by social bots. In other words, each amount of sharing activity by likely bots tends to trigger a disproportionate amount of human engagement. The same amplification effect is not observed for articles from fact-checking sources. Details are presented in Supplementary Discussion (Supplementary Figs. 8, 9).

**Fig. 5:**
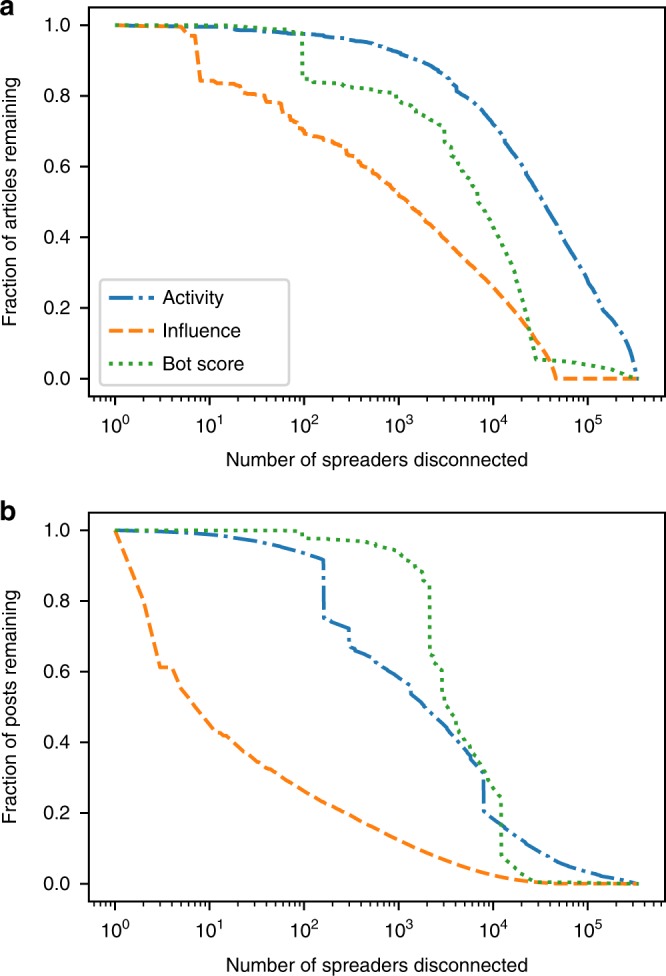
Dismantling the low-credibility content diffusion network. This analysis is based on a network of retweets linking to low-credibility articles, collected during the 2016 US presidential campaign. The network has 227,363 nodes (accounts); see Methods for further details. The priority of disconnected nodes is determined by ranking accounts on the basis of the different characteristics shown in the legend. The remaining fraction of **a** unique articles from low-credibility sources and **b** retweets linking to those articles is plotted versus the number of disconnected nodes

Another way to assess the impact of bots in the spread of low-credibility content is to examine their critical role within the diffusion network. Let us focus on the retweet network^33^, where nodes are accounts and connections represents retweets of messages with links to stories—just like the networks in Fig. 1b, c, but aggregating across all articles from low-credibility sources. We apply a network dismantling procedure^34^: we disconnect one node at a time and analyze the resulting decrease in the total volume of retweets and in the total number of unique articles. The more these quantities are reduced by disconnecting a small number of nodes, the more critical those nodes are in the network. We prioritize accounts to disconnect based on bot score and, for comparison, also based on retweeting activity and influence. Further details can be found in the Methods. Unsurprisingly, Fig. 5 shows that influential nodes are most critical. The most influential nodes are unlikely to be bots, however. Disconnecting nodes with high bot score is the second-best strategy for reducing low-credibility articles (Fig. 5a). For reducing overall post volume, this strategy performs well when about 10% of nodes are disconnected (Fig. 5b). Disconnecting active nodes is not as efficient a strategy for reducing low-credibility articles. These results show that bots are critical in the diffusion network, and that targeting them would significantly improve the quality of information in the network. The spread of links to low-credibility content can be virtually eliminated by disconnecting a small percentage of accounts that are most likely to be bots.

**Fig. 6:**
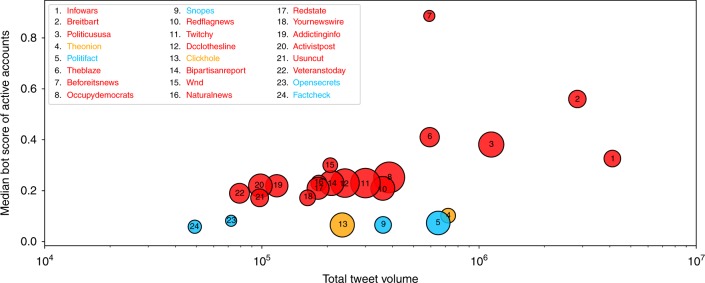
Popularity and bot support for the top sources. Satire websites are shown in orange, fact-checking sites in blue, and low-credibility sources in red. Popularity is measured by total tweet volume (horizontal axis) and median number of tweets per article (circle area). Bot support is gauged by the median bot score of the 100 most active accounts posting links to articles from each source (vertical axis). Low-credibility sources have greater support by bots, as well as greater median and/or total volume in many cases

Finally, we compared the extent to which social bots disseminate content from different low-credibility sources. We considered the most popular sources in terms of median and aggregate article posts, and measured the bot scores of the accounts that most actively spread their content. As shown in Fig. 6, one site (beforeitsnews.com) stands out for the high degree of automation, but other popular low-credibility sources also have many likely bots among their promoters. The dissemination of content from satire sites like The Onion and fact-checking websites does not display the same level of automation; it appears to be more organic.

## Discussion

Our analysis provides quantitative empirical evidence of the key role played by social bots in the spread of low-credibility content. Relatively few accounts are responsible for a large share of the traffic that carries misinformation. These accounts are likely bots, and we uncovered two manipulation strategies they use. First, bots are particularly active in amplifying content in the very early spreading moments, before an article goes “viral.” Second, bots target influential users through replies and mentions. People are vulnerable to these kinds of manipulation, in the sense that they retweet bots who post low-credibility content almost as much as they retweet other humans. As a result, bots amplify the reach of low-credibility content, to the point that it is statistically indistinguishable from that of fact-checking articles. Successful low-credibility sources in the United States, including those on both ends of the political spectrum, are heavily supported by social bots. Social media platforms are beginning to acknowledge these problems and deploy countermeasures, although their effectiveness is hard to evaluate^11,25,35^.

The present findings complement the recent work by Vosoughi et al.^2^ who argue that bots alone do not entirely explain the success of false news. Their analysis is based on a small subset of articles that are fact-checked, whereas the present work considers a much broader set of articles from low-credibility sources, most of which are not fact-checked. In addition, the analysis of Vosoughi et al.^2^ does not consider an important mechanism by which bots can amplify the spread of an article, namely, by resharing links originally posted by human accounts. Because of these two methodological differences, the present analysis provides new evidence about the role played by bots.

Our results are robust with respect to various choices. First, using a more restrictive criterion for selecting low-credibility sources, based on a consensus among several news and fact-checking organizations (see Methods), yields qualitatively similar results, leading to the same conclusions. In addition, the findings are not driven by any single source associated with a large portion of the tweet volume. Second, our analysis about active spreaders of low-credibility content being likely bots is robust with respect to the activity threshold used to identify the most active spreaders. Furthermore, bot scores are uncorrelated with account activity volume. Third, the conclusions are not affected by the use of different bot score thresholds to separate social bots and human accounts. Details about all of these robustness analyses can be found in the Supplementary Discussion (Supplementary Figs. 10–15).

Our findings demonstrate that social bots are an effective tool to manipulate social media. While the present study focuses on the spread of low-credibility content, such as false news, conspiracy theories, and junk science, similar bot strategies may be used to spread other types of malicious content, such as malware. Although our spreading data are collected from Twitter, there is no reason to believe that the same kind of abuse is not taking place on other digital platforms as well. In fact, viral conspiracy theories spread on Facebook^36^ among the followers of pages that, like social bots, can easily be managed automatically and anonymously. While the difficulty to access spreading data on platforms like Facebook is a concern, the growing popularity of ephemeral social media like Snapchat may make future studies of this type of abuse all but impossible.

The results presented here suggest that curbing social bots may be an effective strategy for mitigating the spread of low-credibility content, and that the bot score might provide a useful signal to prioritize accounts for further review. Progress in this direction may be accelerated through partnerships between social media platforms and academic research^11^. For example, our lab and others are developing machine learning algorithms to detect social bots^10,26,27^. The deployment of such tools is fraught with peril, however. While platforms have the right to enforce their terms of service, which forbid impersonation and deception, algorithms do make mistakes. Even a single false-positive error leading to the suspension of a legitimate account may foster valid concerns about censorship. This justifies current human-in-the-loop solutions which unfortunately do not scale with the volume of abuse that is enabled by software. It is therefore imperative to support research both on improved abuse detection algorithms and on countermeasures that take into account the complex interplay between the cognitive and technological factors that favor the spread of misinformation^37^.

An alternative strategy would be to employ CAPTCHAs^38^, challenge–response tests to determine whether a user is human. CAPTCHAs have been deployed widely and successfully to combat email spam and other types of online abuse. Their use to limit automatic posting or resharing of news links could help stem bot abuse by increasing its cost, but also add undesirable friction to benign applications of automation by legitimate entities, such as news media and emergency response coordinators. These are hard trade-offs that must be studied carefully as we contemplate ways to address the fake news epidemics.

The present study focuses on the role of social bots in the spread of low-credibility content. These kinds of bots are often deceptive. For example, none of the ten Twitter accounts most active at retweeting articles in the core of the misinformation network during the study period identified themselves as bots^33^. One question that has not been addressed is whether similar patterns of amplification might be observed in the spread of content from legitimate, high-quality news sources. Mainstream media do use automated accounts to post news feeds, although these bots do not deceptively impersonate humans. While preliminary analysis suggests that mainstream media do not display the same systematic bot support observed for low-credibility sources (Supplementary Fig. 8), the use of bots to promote legitimate news content deserves further investigation.

Finally, the present study focuses on US sources during the period preceding and following the 2016 US presidential election. It will be important to explore whether bot manipulation of social media platforms is concentrated around major electoral events in the United States and other countries.

## Methods

### Hoaxy data

Data about articles shared on Twitter were collected through Hoaxy, an open platform developed at Indiana University to track the spread of claims and fact checking^33^. A search engine, interactive visualizations, and open-source software are freely available (hoaxy.iuni.iu.edu). The data are accessible through a public application program interface (API). Further details are presented in Supplementary Methods.

The collection period for the present analysis extends from mid-May 2016 until the end of March 2017. During this time, we collected 389,569 articles from 120 low-credibility sites. We also tracked 15,053 stories published by independent fact-checking organizations, such as snopes.com, politifact.com, and factcheck.org.

The list of low-credibility sources was obtained by merging several lists compiled by third-party news and fact-checking organizations or experts. The collection started with 71 sites and 49 more were added in mid-December 2016. The full list of sources and their provenance is reported in Supplementary Table 1. Many low-credibility sources label their content as satirical, and viral satire is sometimes mistaken for real news. For these reasons, satire sites are not excluded from the list of low-credibility sources. However, our findings are robust with respect to this choice. The Onion is the satirical source with the highest total volume of shares. We repeated our analyses of most viral articles (e.g., Fig. 3a) with articles from theonion.com excluded and the results were not affected.

We also repeated the analysis using a more restrictive criterion for selecting low-credibility sources, based on a consensus among three or more news and fact-checking organizations. This yields 327,840 articles (86% of the total) from 65 low-credibility sources, also listed in Supplementary Methods, where we show that the results are robust with respect to these different source selection criteria.

Our analysis does not require a complete list of low-credibility sources, but does rely on the assumption that many articles published by these sources can be classified as some kind of misinformation or unsubstantiated information. To validate this assumption, we checked the content of a random sample of articles. For the purpose of this verification, we adopted a definition of “misinformation” that follows industry convention and includes the following classes: fabricated content, manipulated content, imposter content, false context, misleading content, false connection, and satire^39^. To these seven categories we also added articles whose claims could not be verified. We found that fewer that 15% of articles could be verified. More details are available in Supplementary Methods (Supplementary Figs. 3, 4).

Using the filtering endpoint of Twitter’s public streaming API, we collected 13,617,425 public posts that included links to articles from low-credibility sources and 1,133,674 public posts linking to fact checks. This is the complete set of tweets linking to these articles in the study period, and not a sample (see Supplementary Methods for details). We extracted metadata about the source of each link, the account that shared it, the original poster in case of retweet or quoted tweet, and any users mentioned or replied to in the tweet.

We transformed links to canonical URLs to merge different links referring to the same article. This happens mainly due to shortening services (44% links are redirected) and extra parameters (34% of URLs contain analytics tracking parameters), but we also found websites that use duplicate domains and snapshot services. Canonical URLs were obtained by resolving redirection and removing analytics parameters.

In the targeting analysis (Fig. 3b), we exclude mentions of sources using the pattern “via @screen_name.”

### Botometer scores

The bot score of Twitter accounts is computed using the Botometer classifier which evaluates the extent to which an account exhibits similarity to the characteristics of social bots^26^. The system is based on a supervised machine learning algorithm leveraging more than a thousand features extracted from public data and metadata about Twitter accounts. These features include various descriptors of information diffusion networks, user metadata, friend statistics, temporal patterns of activity, part-of-speech, and sentiment analysis. The classifier is trained using publicly available datasets of tens of thousands of Twitter users that include both humans and bots of varying sophistication. The Botometer system is available through a public API (botometer.iuni.iu.edu). It has also been employed in other studies^2,40^ and is widely adopted, serving hundreds of thousand requests daily.

For the present analysis, we use the Twitter Search API to collect up to 200 of an account’s most recent tweets and up to 100 of the most recent tweets mentioning the account. From these data we extract the features used by the Botometer classifier. We use logistic calibration to make the bot scores calculated by the classifier easier to interpret as confidence levels (see Supplementary Methods and Supplementary Fig. 5).

There are many types of bots and humans using different levels of automation. Accordingly, Botometer provides a score rather than a binary classification. Nevertheless, the model can effectively discriminate between human and bot accounts of different nature; fivefold cross-validation yields an area under the receiver operating characteristic curve (AUC) of 94%^26^. (An AUC value of 50% indicates random accuracy and 100% means perfect accuracy.) When a binary classification is needed, we use a bot score threshold of 0.5 which maximizes accuracy^26^. See Supplementary Methods and Discussion for further details about bot classification and the robustness of results based on a bot score threshold.

### Retweet network

The network studied in the dismantling analysis (Fig. 5) is based on retweets with links to articles from low-credibility sources, posted before the 2016 US presidential election (16 May–7 November 2016). The network has 227,363 nodes (accounts) and 816,453 directed edges. Each edge is weighted by the number of retweets between the same pair of accounts. When an account is disconnected, all of its incoming and outgoing edges are removed. When we disconnect a retweeting node *i* that was in turn retweeted by some node *j*, only *i* is removed because in the Twitter metadata, each retweet connects directly to the account that originated the tweet. Given the directionality of edges, retweeting activity is measured by node in-strength centrality (weighted in-degree) and influence by out-strength centrality (weighted out-degree).

### Code availability

Code used to carry out the analyses in this manuscript is available on Github (github.com/IUNetSci/HoaxyBots). Hoaxy is an open-source project and all system software is public (github.com/IUNetSci). Reasonable additional requests and questions about code can be directed to the corresponding author.

## Electronic supplementary material

Supplementary Information

## Data Availability

There are two data sources analyzed during the current study: Hoaxy for data about article diffusion, and Botometer for data about Twitter bot scores. Further details are available in Supplementary Methods. Datasets used to carry out the analyses in this manuscript are available on Zenodo (10.5281/zenodo.1402267). Additionally, data about article diffusion and bot scores are available through the public Hoaxy API (hoaxy.iuni.iu.edu) and the public Botometer API (botometer.iuni.iu.edu), respectively. Reasonable additional requests and questions about data or APIs can be directed to the corresponding author.
