# Supplementary material for: The spread of low-credibility content by social bots
Source: Nat Commun. 2018 Nov 20;9:4787. doi: 10.1038/s41467-018-06930-7 (PMC6246561; doi:10.1038/s41467-018-06930-7)
Supplement: Supplementary file 1 — Supplementary Information [file 41467_2018_6930_MOESM1_ESM.pdf]

The spread of low-credibility content by social bots

Supplementary Information

Shao *et al.*

Supplementary Methods

List of Sources Our list of low-credibility sources was obtained by merging several lists compiled by third-party news and fact-checking organizations or experts. It should be noted that these lists were compiled independently of each other, and as a result they have uneven coverage. However, there is overlap between them. The full list of sources and their provenance are shown in Supple-mentary Table 1. Some lists annotate sources in different categories. In the case of OpenSources (`www.opensources.co`[1]), we only considered sources tagged with any of the following labels: *fake, satire, bias, conspiracy, rumor, state, junksci, clickbait, hate.* In the case of Starbird's list of alternative domains[1], we considered those with primary orientation coded as one of *conspiracy theorists, political agenda, tabloid—clickbait news.* Some lists (labeled KS, BF, and PF in the table) were not yet available or known to us when we started the collection. The sources in those

[1] We extracted sources from this list in Spring 2016.

lists where added in December 2016. At that time some of the existing lists, such as OpenSources, had been expanded, therefore some additional sources were added as a result of those updates as well, bringing the total to 120 sources.

For robustness analysis (below), we also consider a "consensus" subset of sites that are each listed among low-credibility sources by at least three organizations or experts. This subset includes 65 sources, also shown in Supplementary Table 1. We track 10,663,818 tweets (79% of the total) with links to 327,840 articles (86% of the total) from consensus low-credibility source, generated by 1,135,167 accounts (84% of the total).

We additionally tracked the websites of seven independent fact-checking organizations:

1. `politifact.com`
2. `snopes.com`
3. `factcheck.org`
4. `opensecrets.org`
5. `hoax-slayer.com`[2]
6. `badsatiretoday.com`
7. `truthorfiction.com`

[2] `hoax-slayer.com` includes its older version `hoax-slayer.net`.

Supplementary Table 1: Low-credibility sources. For each source, we indicate which lists include it. The lists are: Fake News Watch (FNW), OpenSources (OS), Daily Dot (DD), US News & World Report (US), New Republic (NR), CBS, Urban Legends (UL), NPR, Snopes Field Guide (Sn), Starbird Alternative Domains (KS), BuzzFeed News (BF), and PolitiFact (PF). Table headers link to the original lists. The date indicates when Hoaxy started following a source: May or December 2016. Consensus sources (in three or more lists) are shown in italics.

Source	FNW	OS	DD	US	NR	CBS	UL	NPR	Sn	KS	BF	PF	Date
21stcenturywire.com	✓	✓	✓							✓			May
70news.wordpress.com		✓	✓			✓							Dec
abcnews.com.co		✓	✓			✓					✓	✓	Dec
activistpost.com	✓	✓	✓	✓						✓			May
addictinginfo.org		✓	✓							✓			Dec
americannews.com	✓	✓	✓	✓								✓	May
americannewsx.com		✓											Dec
amplifyingglass.com	✓												May
anonews.co			✓										Dec
beforeitsnews.com	✓	✓		✓						✓		✓	May
bigamericannews.com	✓	✓											May
bipartisanreport.com		✓	✓										Dec
bluenationreview.com		✓	✓										Dec
breitbart.com		✓	✓							✓			Dec
burrardstreetjournal.com		✓				✓					✓		Dec
callthecops.net		✓	✓				✓						Dec
christiantimes.com						✓							Dec
christwire.org	✓	✓	✓										May
chronicle.su	✓	✓											May
civictribune.com	✓	✓	✓			✓					✓	✓	May
clickhole.com	✓	✓	✓	✓									May
coasttocoastam.com	✓		✓										May
collective-evolution.com			✓										Dec
consciouslifenews.com	✓	✓	✓							✓			May
conservativeoutfitters.com	✓	✓	✓										Dec
countdowntozerotime.com	✓	✓	✓										May
counterpsyops.com	✓	✓											May
creambmp.com	✓	✓	✓										Dec
dailybuzzlive.com	✓	✓		✓								✓	May
dailycurrant.com	✓	✓					✓				✓		May
dailynewsbin.com		✓											Dec
dcclothesline.com	✓	✓								✓			May
demyx.com					✓								Dec
denverguardian.com		✓						✓			✓		Dec
derfmagazine.com	✓	✓											May
disclose.tv	✓	✓		✓								✓	May
duffelblog.com	✓		✓	✓								✓	May
duhprogressive.com	✓	✓											May
empireherald.com		✓				✓					✓	✓	Dec
empirenews.net	✓	✓	✓			✓	✓		✓		✓	✓	May
empiresports.co	✓	✓			✓		✓		✓		✓	✓	May

Continued on next page

Supplementary Table 1 – continued from previous page

Source	FNW	OS	DD	US	NR	CBS	UL	NPR	Sn	KS	BF	PF	Date
en.mediamass.net	✓		✓		✓		✓						Dec
endingthefed.com		✓											Dec
enduringvision.com	✓	✓	✓										May
flyheight.com		✓											Dec
fprnradio.com	✓	✓											May
freewoodpost.com		✓					✓				✓	✓	Dec
geoengineeringwatch.org	✓	✓											May
globalassociatednews.com					✓		✓				✓		Dec
globalresearch.ca	✓	✓								✓			May
gomerblog.com	✓												May
govtslaves.info	✓	✓								✓			May
gulagbound.com	✓	✓											May
hangthebankers.com	✓	✓											May
humansarefree.com	✓	✓											May
huzlers.com	✓	✓			✓	✓	✓		✓		✓	✓	May
ifyouonlynews.com		✓				✓							Dec
infowars.com	✓	✓	✓	✓		✓				✓			May
intellihub.com	✓	✓								✓			May
itaglive.com	✓												May
jonesreport.com	✓	✓											Dec
lewrockwell.com	✓	✓								✓			May
liberalamerica.org		✓											Dec
libertymovementradio.com	✓	✓											May
libertytalk.fm	✓	✓											May
libertyvideos.org	✓	✓											Dec
lightlybraisedturnip.com					✓								Dec
nationalreport.net	✓	✓	✓		✓	✓	✓	✓	✓		✓	✓	May
naturalnews.com	✓	✓	✓	✓									May
ncscooper.com		✓							✓		✓		Dec
newsbiscuit.com	✓	✓	✓								✓		May
newslo.com[a]	✓	✓	✓	✓							✓	✓	May
newsmutiny.com	✓	✓	✓										May
newswire-24.com	✓	✓											May
nodisinfo.com	✓	✓								✓			May
now8news.com		✓				✓			✓		✓	✓	Dec
nowtheendbegins.com	✓	✓											May
occupydemocrats.com		✓	✓							✓			Dec
other98.com		✓	✓										Dec
pakalertpress.com	✓	✓											May
politicalblindspot.com	✓	✓											May
politicalears.com	✓	✓											May
politicops.com[a]	✓	✓				✓					✓	✓	May
politicususa.com		✓											Dec
prisonplanet.com	✓	✓											May
react365.com		✓				✓			✓		✓	✓	Dec
realfarmacy.com	✓	✓											May
realnewsrightnow.com	✓	✓	✓			✓					✓	✓	May
redflagnews.com	✓	✓		✓						✓			May
redstate.com		✓	✓										Dec

Continued on next page

Supplementary Table 1 – continued from previous page

Source	FNW	OS	DD	US	NR	CBS	UL	NPR	Sn	KS	BF	PF	Date
rilenews.com	✓	✓	✓			✓					✓		May
rockcitytimes.com	✓												May
satiratribune.com		✓							✓		✓	✓	Dec
stuppid.com		✓							✓		✓		Dec
theblaze.com		✓											Dec
thebostontribune.com		✓				✓					✓		Dec
thedailysheeple.com	✓	✓								✓			May
thedcgazette.com[b]	✓		✓	✓		✓							May
thefreethoughtproject.com		✓	✓							✓			Dec
thelapine.ca	✓						✓						May
thenewsnerd.com	✓	✓			✓						✓		May
theonion.com	✓	✓	✓	✓	✓	✓	✓						Dec
theracketreport.com		✓					✓				✓	✓	Dec
therundownlive.com	✓	✓											May
thespoof.com	✓	✓					✓						May
theuspatriot.com	✓	✓											May
truthfrequencyradio.com	✓	✓											Dec
twitchy.com			✓										Dec
unconfirmedsources.com	✓	✓											May
usuncut.com		✓	✓										Dec
veteranstoday.com	✓	✓								✓			May
wakingupwisconsin.com	✓	✓											May
weeklyworldnews.com	✓	✓		✓			✓						May
wideawakeamerica.com	✓												Dec
winningdemocrats.com		✓											Dec
witscience.org	✓	✓									✓		May
wnd.com		✓											Dec
worldnewsdailyreport.com	✓	✓	✓				✓		✓		✓	✓	May
worldtruth.tv	✓	✓		✓						✓		✓	May
yournewswire.com		✓				✓				✓	✓	✓	Dec

[a] `newslo.com` and `politicops.com` are mirrors of `politicot.com`.

[b] `thedcgazette.com` is a mirror of `dcgazette.com`.

Hoaxy Data The back-end component of Hoaxy collects public tweets that link to a predefined list of websites. We use the "POST statuses/filter" endpoint of the Twitter streaming API, which filters the real-time stream of public tweets ("firehose") in such a way as to provide tweets that match a standing query. Our query includes the domain names of all tracked sources. Tweets with links to these sources match the query, even if URLs are shortened. This methodology, together with the

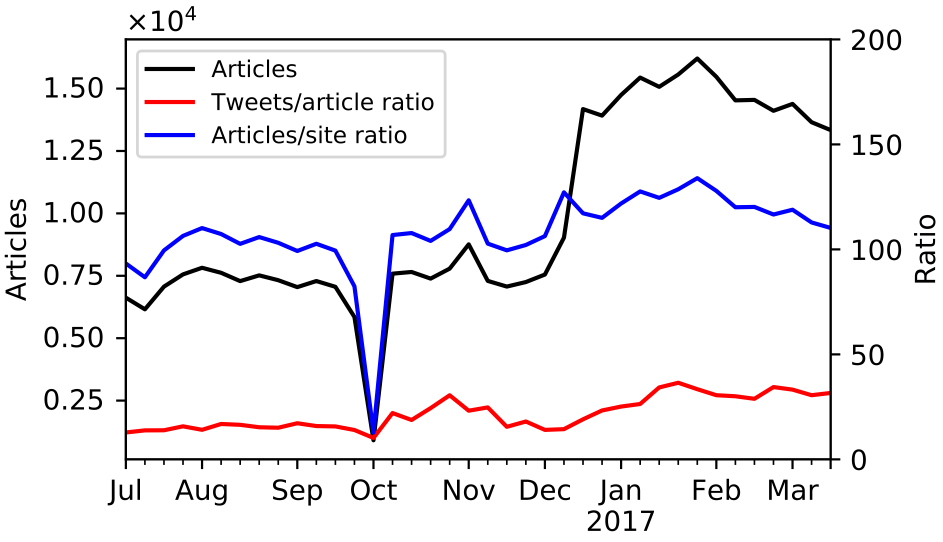

Supplementary Figure 1: Weekly tweeted low-credibility articles, tweets/article ratio and articles/site ratio. The collection was briefly interrupted in October 2016. In December 2016 the set of low-credibility sources was expanded from 71 to 120 websites.

fact that the total volume of tweets collected is well below 1% of all public tweets, guarantees that we obtain *all* tweets linking to the sites in our list, and not a sample of the tweets with these links.

In addition, Hoaxy crawls all tracked websites and indexes all their articles, supporting a full-text search engine that allows users to find articles matching a given query. Furthermore, users can select subsets of these articles to visualize their spread on Twitter. To this end, Hoaxy matches the indexed articles with the tweets in our database and constructs networks based on retweets, mentions, replies, and quoted tweets. The front-end visualizes these networks interactively, allowing users to explore the accounts (nodes) and the tweets (edges) that make up these networks. The system makes all the data accessible to the public through a website (`hoaxy.iuni.iu.edu`) and an API.

Our analysis focuses on the period from mid-May 2016 to the end of March 2017. During this time, we collected 15,053 and 389,569 articles from fact-checking and low-credibility sources, respectively. The Hoaxy system collected 1,133,674 public posts that included links to fact checks and 13,617,425 public posts linking to low-credibility articles. As shown in Supplementary Fig. 1, low-credibility websites each produced approximately 100 articles per week, on average. Toward the end of the study period, this content was shared by approximately 30 tweets per article per week, on average. However, as discussed in the main text, success is extremely heterogeneous across articles. This is the case irrespective of whether we measure success through the number of tweets (Supplementary Fig. 2(a), also in main text) or accounts (Supplementary Fig. 2(b)) sharing an article. For both popularity measures, the distributions are very broad and basically indistinguishable across articles from low-credibility vs. fact-checking sources.

Content Analysis Our analysis considers content published by a set of websites flagged as sources of misinformation by third-party journalistic and fact-checking organizations (Supplementary Table 1). This source-based approach relies on the assumption that most of the articles published by our compilation of sources are some type of misinformation, as we cannot fact-check each individual article. We validated this assumption by estimating the rate of false positives, i.e, verified articles, in the corpus. We manually evaluated a random sample of articles ($N = 50$) drawn from our corpus, stratified by source. We considered only those sources whose articles were tweeted at least once in the period of interest. To draw an article, we first selected a source at random with replacement, and then chose one of the articles it published, again at random but without replacement. We repeated our analysis on an additional sample ($N = 50$) in which the chances of drawing

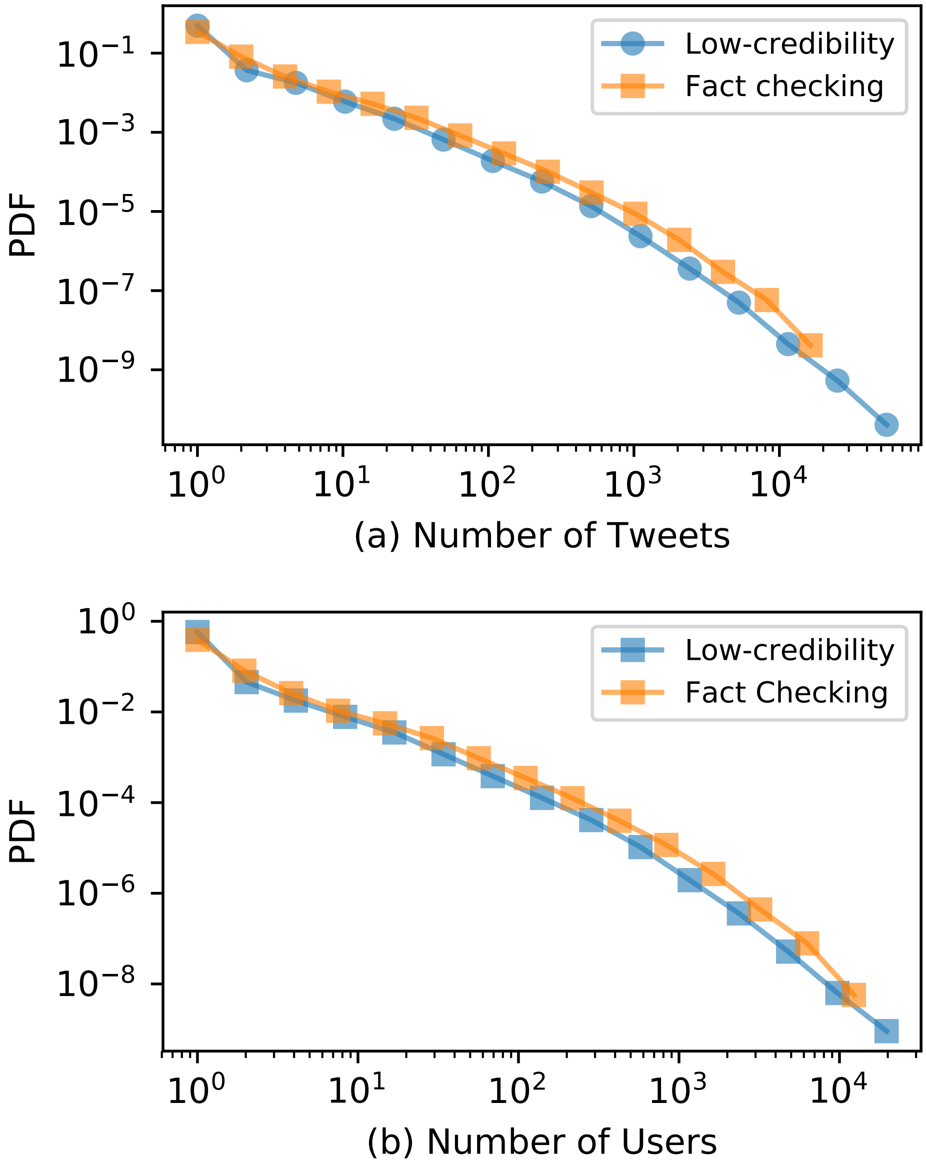

Supplementary Figure 2: Probability distributions of popularity of articles from low-credibility and fact-checking sources, measured by (a) the number of tweets and (b) the number of accounts sharing links to an article.

an article are proportional to the number of times it was tweeted. This 'sample by tweet' is thus biased toward more popular sources.

It is important to note that articles with unverified claims are sometimes updated after being debunked. This happens usually late, after the article has spread, and could lead to overestimating the rate of false positives. To mitigate this phenomenon, the earliest snapshot of each article was retrieved from the Wayback Machine at the Internet Archive (`archive.org`). If no snapshot was available, we retrieved the version of the page current at verification time. If the page was missing from the website or the website was down, we reviewed the title and body of the article crawled by Hoaxy. We gave priority to the current version over the possibly more accurate crawled version because, in deciding whether a piece of content is misinformation, we want to consider any form of visual evidence included with it, such as images or videos.

After retrieving all articles in the two samples, each article was evaluated independently by two reviewers (two of the authors), using a rubric summarized in Supplementary Fig. 3. Each article was then labeled with the majority label, with ties broken by a third reviewer (another author). Supplementary Fig. 4 shows the results of the analysis. We report the fractions of articles that were verified and that could not be verified (inconclusive), out of the total number of articles that contain any factual claim. The rate of false positives is below 15% in both samples.

Concentration In the main text we use the Gini coefficient to calculate the concentration of posting activity for an article, based on the accounts that post links to the article. For each article, the Lorenz curve shows the cumulative share of tweets versus the cumulative share of accounts gener-

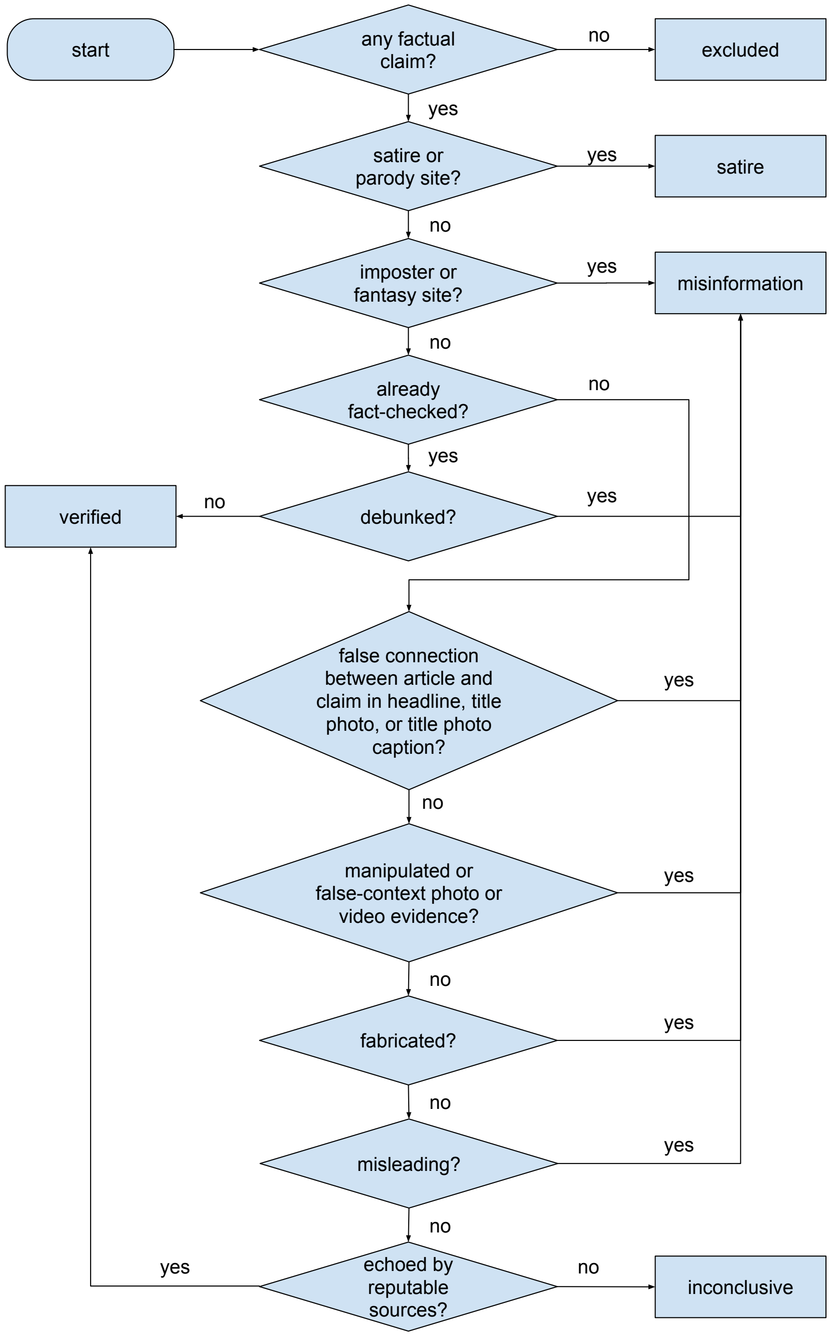

Supplementary Figure 3: Flowchart summarizing the annotation rubric employed in the content analysis.

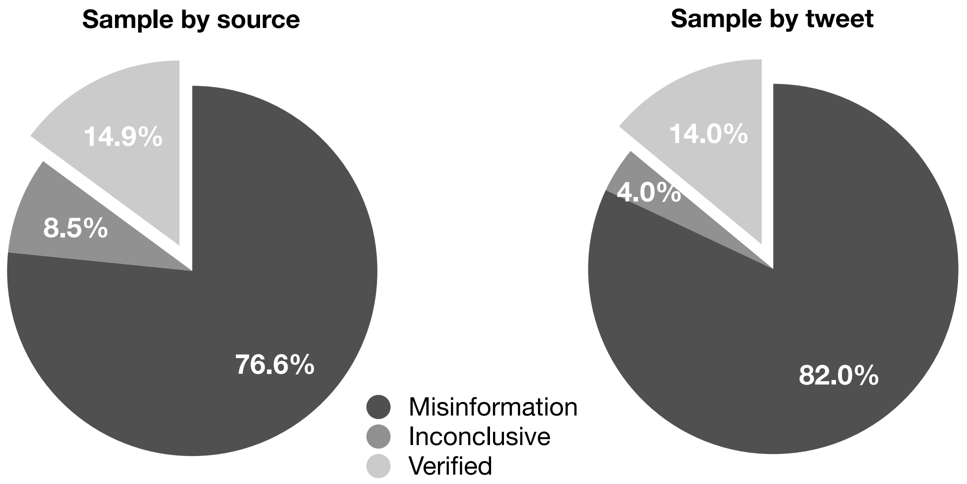

Supplementary Figure 4: Content analysis based on two samples of articles. Sampling by source gives each source equal representation, while sampling by tweets biases the analysis toward more popular sources. We excluded from the sample by source three articles that did not contain any factual claims. Satire articles are grouped with misinformation, as explained in the main text.

ating these tweets. The Gini coefficient is the ratio of the area that lies between the line of equality (diagonal) and the Lorenz curve, over the total area under the line of equality. A high coefficient indicates that a small subset of accounts was responsible for a large portion of the posts.

Bot Score Calibration Calibration methods are applicable when a machine learning classifier outputs probabilistic scores. Well-calibrated classifiers are probabilistic models for which the estimates can be directly interpreted as confidence levels. We use Platt's scaling[2], a logistic regression model trained on classifier outputs, to calibrate the bot score computed by the Botometer classifier.

We present the mapping between raw and calibrated scores in Supplementary Fig. 5. The calibration only changes scores within the unit interval, but retains the ranking among accounts. The figure also shows reliability diagrams for raw and calibrated scores[3]. We split the unit interval into 20 bins. Each instance in the training data set is assigned to a bin based on its predicted (raw)

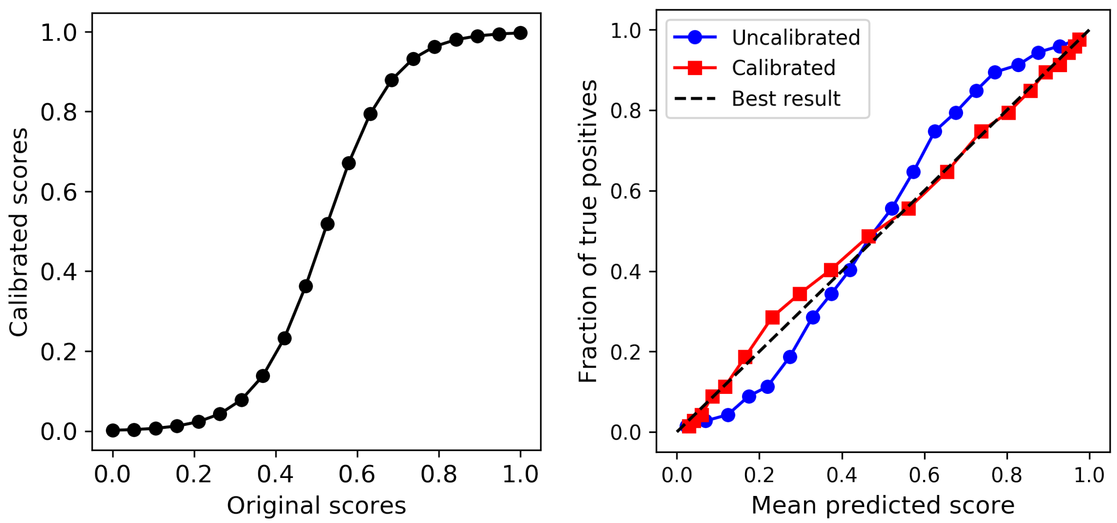

Supplementary Figure 5: Bot score calibration curves. Left: The calibration mapping function projects raw classifier output to calibrated scores. Right: Reliability curves show the true positive rate against the mean predicted scores. The calibrated curve indicates higher reliability because it is closer to the unbiased diagonal line.

score. For each bin, the mean predicted score is computed and compared against the fraction of true positive cases. In a well-calibrated model, the points align to the diagonal.

Bot Classification To show that a few social bots are disproportionately responsible for the spread of low-credibility content, we considered a random sample of accounts that shared at least one article from a low-credibility source, and evaluated these accounts using the bot classification system Botometer. Out of 1,000 sampled accounts, 85 could not be inspected because they had been either suspended, deleted, or turned private. For each of the remaining 915, Botometer returned a *bot score* estimating the level of automation of the account. To quantify how many accounts are likely bots, we transform bot scores into binary assessments using a threshold of 0.5. This is a conservative choice to minimize false negatives and especially false positives, as shown in prior work (cit. in main text). Supplementary Table 2 shows the fraction of accounts with scores above

the threshold. To give a sense of their overall impact in the spreading of low-credibility content, Supplementary Table 2 also shows the fraction of tweets with articles from low-credibility sources posted by accounts that are likely bots, and the number of unique articles included in those tweets overall. As a comparison, we also tally the fact-checks shared by these accounts, showing that bot accounts focused on sharing low-credibility content and ignored fact-checking content.

In the main text we show the distributions of bot scores for this sample of accounts, as well as for a sample of accounts that were most active in spreading low-credibility content (*super-spreaders*). To select the super-spreaders, we ranked all accounts by how many tweets they posted with links to low-credibility sources, and considered the top 1,000 accounts. We then performed the same classification steps discussed above. For the same reasons mentioned above, we could not obtain scores for 39 of these accounts, leaving us with a sample of 961 scored accounts. We experimented with different activity thresholds and found that they do not change our conclusions that super-spreaders are more likely to be social bots.

Supplementary Discussion

Super-Spreaders of Low-Credibility Content In the main text we show that the more popular a low-credibility article, the more its posting activity is concentrated around a relative small number of active accounts. We also find that the most active spreaders of content from low-credibility sources are more likely to be social bots. To further illustrate the anomalous activity patterns of these "super-spreaders," Supplementary Fig. 6 shows the distribution of repeated tweets by

Supplementary Table 2: Analysis of likely bots and their content spreading activity based on a random sample of Twitter accounts sharing at least one article from a low-credibility source.

	Total	Likely bots	Percentage
Accounts	915	54	6%
Tweets with low-credibility articles	11,656	3,587	31%
Unique low-credibility articles	7,726	2,608	34%
Tweets with fact-checks	598	4	0.7%
Unique fact-checks	395	3	0.8%

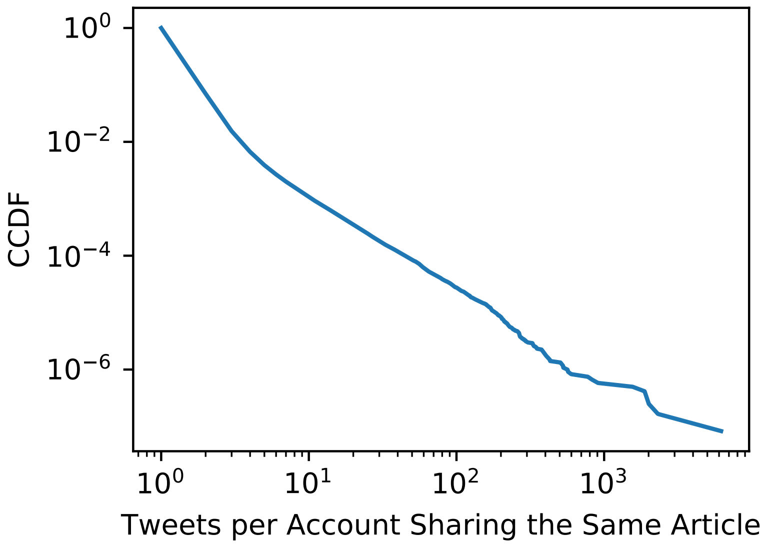

Supplementary Figure 6: Complementary cumulative distribution of repetitions, i.e., the number of times a single account tweets the same link to an article from a low-credibility source.

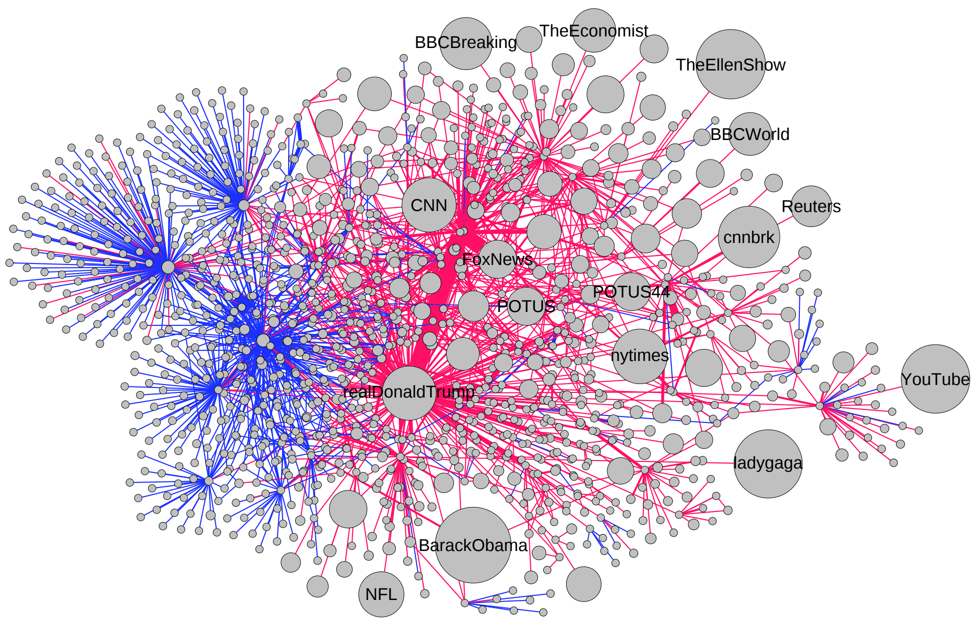

Supplementary Figure 7: Example of targeting for the article *Report: three million votes in presidential election cast by illegal aliens*, published by `InfoWars.com` on November 14, 2016 and shared over 18 thousand times on Twitter. Only a portion of the diffusion network is shown. Nodes stand for Twitter accounts, with size representing number of followers. Links illustrate how the article spreads: by retweets and quoted tweets (blue), or by replies and mentions (red). The thickest red link connecting to the `@realDonaldTrump` node corresponds to the case described in the text.

individual accounts sharing the same low-credibility article. While it is normal behavior for a person to share an article once, the long tail of the distribution highlights inorganic, automated support. A single account posting the same article over and over — hundreds or thousands of times in some cases — is likely controlled by software.

Bots Targeting Influentials The main text discusses a strategy used by bots, by which influential users are mentioned in tweets that link to low-credibility content. Bots seem to employ this targeting strategy repetitively. Supplementary Fig. 7 offers an illustration: in this example,

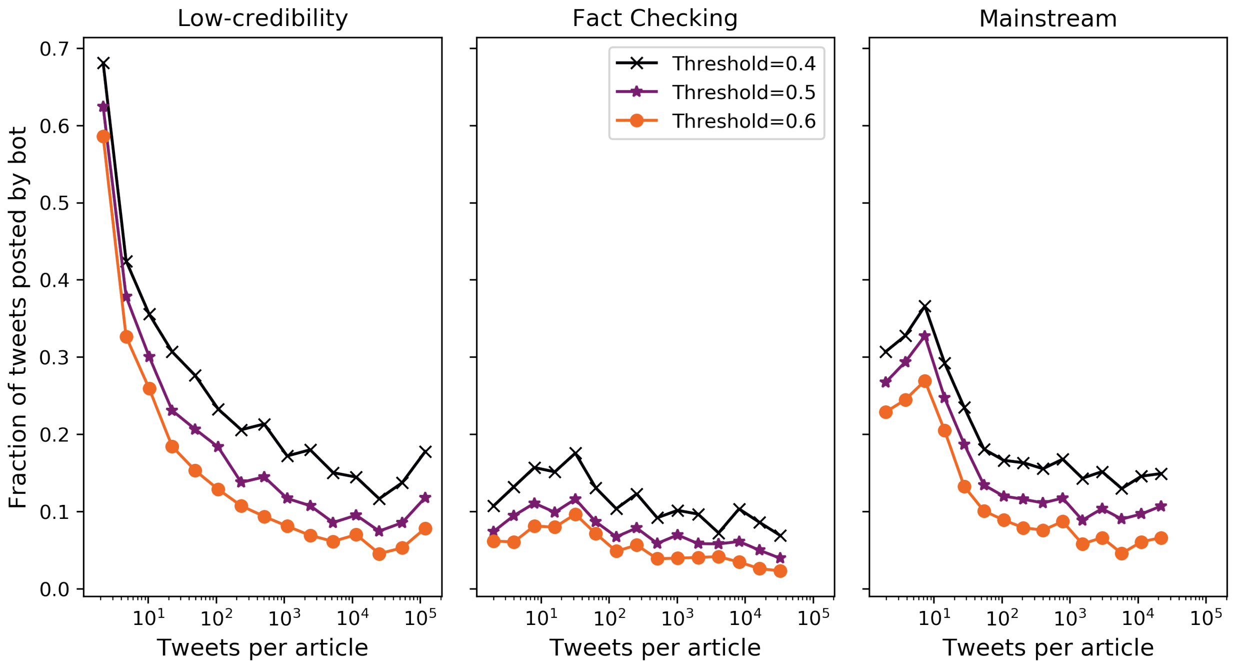

Supplementary Figure 8: Fraction of tweets linking to news articles that are posted by accounts with bot score above a threshold, as a function of the popularity of the linked articles. We see different bot activity for articles from low-credibility (left) versus fact-checking (center) and mainstream (right) sources.

a single account produced 19 tweets linking to the article shown in the figure and mentioning `@realDonaldTrump`.

Amplification by Bots The analysis in the main text focuses on the role of bots in the spread of articles from low-credibility sources, assuming that bots do not equally support the spread of articles from fact-checking sources. In fact, we show in the main text that articles from low-credibility and fact-checking sources spread through different mixes of original tweets, retweets, and replies. And we also find that low-credibility sources have greater support from bots than fact-checking and satire sources. To further confirm the assumption that bots do not play an equal role in the spread of fact-checking articles, we observe in Supplementary Fig. 8 that the fraction

of tweets posted by likely bots is higher for articles from low-credibility sources. The biggest difference in the proportion of tweets from bots is observed among unpopular articles, where bot support is much more visible in the case of articles from low-credibility sources; the support gets diluted for more popular articles. The fraction is flatter for articles from fact-checking sources. This raises the question of whether fact-checking sources provide the best baseline. In fact, even articles from reliable sources may be promoted by automated accounts to some degree — the important difference is that such accounts are not deceptive. For example, the official Twitter account of a mainstream news source may automatically post all stories from that source, without impersonating human users.

To explore the extent to which mainstream news sources may be amplified by bots, we conducted an additional analysis. We collected tweets linking to three top mainstream news sources (*New York Times, USA Today, Los Angeles Times*) for a few days in late July 2018 (2018-07-28 02:26 – 2018-07-31 09:38). This dataset includes 379,471 tweets by 207,207 accounts linking to 22,732 articles (unique URLs). We acknowledge that the time period, number of sources, and volume of our data related to mainstream media are all different from those of the data related to low-credibility and fact-checking sources. With this caveat, the right-hand panel of Supplementary Fig. 8 suggests that mainstream news sources may have more support from automated accounts compared to fact-checking sources. As we consider less popular stories from mainstream sources, the presence of automated accounts is more noticeable. However, for low-popularity articles (below 10 tweets), bot support is not nearly as strong as for low-credibility sources. This suggests systematic amplification of low-credibility sources. Due to the caveat mentioned above,

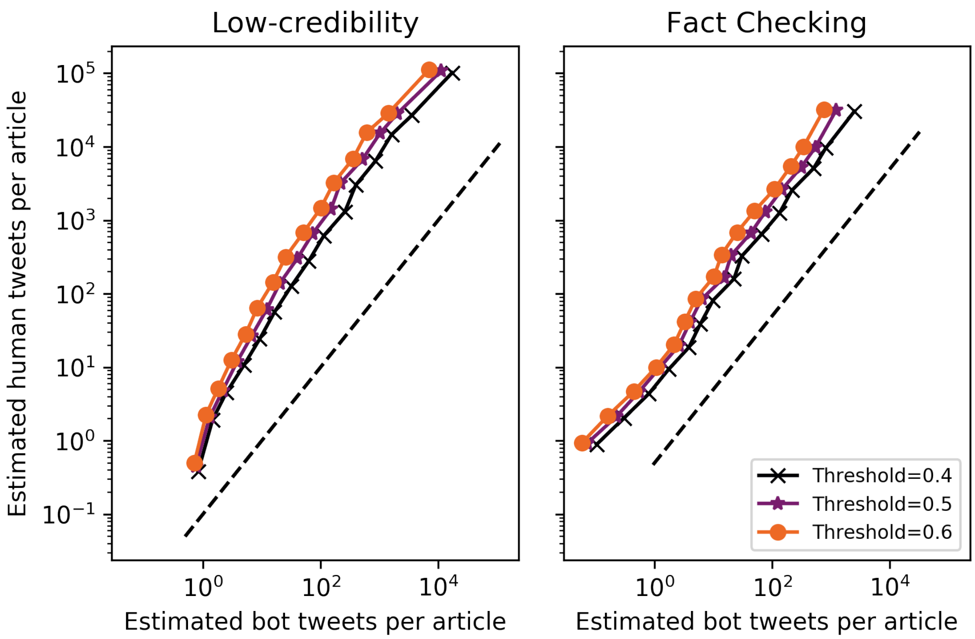

Supplementary Figure 9: For links to articles from low-credibility (left) and fact-checking (right) sources, the number of tweets by accounts with bot score above a threshold is plotted versus the number of tweets by accounts with bot score below the threshold. The dashed lines are guides to the eye, showing linear growth. A super-linear relationship is a signature of amplification by bots.

the question of bot support for mainstream media requires further investigation, as discussed in the main text.

In the analysis of Supplementary Fig. 8, bots and humans are separated based on a threshold in the bot score. These findings are robust to the choice of threshold, and point to selective amplification of articles from low-credibility sources by bots.

To focus on amplification more directly, let us consider how exposure to humans varies with activity by bots. Supplementary Fig. 9 estimates the numbers of tweets by likely humans/bots, using a threshold on bot scores to separate them. Results are robust with respect to the choice of threshold. For articles from low-credibility sources, the estimated number of human tweets per

article grows faster than the estimated number of bot tweets for article. For fact-checking articles, instead, we find a linear relationship. In other words, bots seem to amplify the reach of articles from low-credibility sources, but not the reach of articles from fact-checking sources.

Robustness Analyses The results in the main text are robust with respect to various choices and assumptions, presented next.

Criteria for selection of sources

We repeated the analyses in the main text using the more restrictive criterion for selecting low-credibility sources, based on a consensus among three or more news and fact-checking organizations. The 65 consensus sources are listed in Supplementary Table 1. To carry out these analyses, we inspected 33,115 accounts and could obtain bot scores for 32,250 of them; the rest had been suspended or gone private. The results are qualitatively similar to those in the main text and support the robustness of the findings, namely: super-spreaders of articles from low-credibility sources are likely bots (Supplementary Fig. 10), bots amplify the spread of information from low-credibility sources in the early phases (Supplementary Fig. 11), bots target influential users (Supplementary Fig. 12), and humans retweet low-credibility content posted by bots (Supplementary Fig. 13).

The analysis in Supplementary Fig. 11 is carried out removing tweets with links to *The Onion*, the most popular satire source, to show that the results are also robust with respect to the inclusion/exclusion of satire sites.

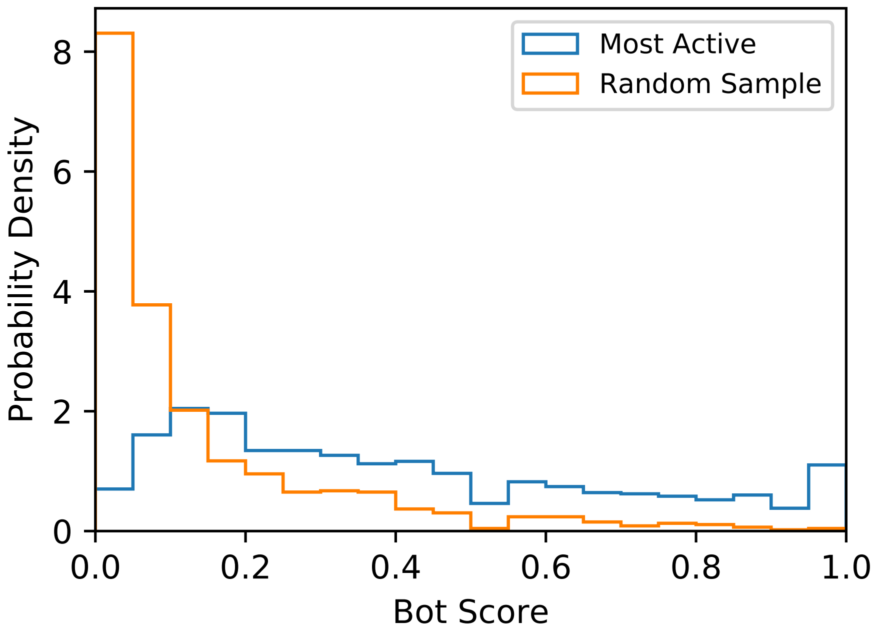

Supplementary Figure 10: Bot score distributions for super-spreaders vs. randomly selected sharers of links to low-credibility sources selected by the consensus criterion. The random sample includes 992 accounts who posted at least one link to an article from a low-credibility source. Their bot scores are compared to 997 accounts that most actively share such links. The two groups have significantly different scores ($p < 10^{-4}$ according to a Mann-Whitney U test).

The peak in correspondence of bot score near 0.5 that can be observed in the left-hand panel of Supplementary Fig. 13 is due to a single account, `@PrisonPlanet`, whose posts linking to low-credibility content are most heavily retweeted. In fact, the account is associated with two of the low-credibility sources, `PrisonPlanet.com` and `Infowars.com`, both controlled by the same entity. If we remove the retweets of `@PrisonPlanet`, we obtain the distributions shown in Supplementary Fig. 14. The next two peaks in correspondence of bot scores between 0.3 and 0.4 are due to two other accounts associated with low-credibility sources, namely `@RealAlexJones` (owner of `PrisonPlanet.com` and `Infowars.com`) and `@TheOnion`. Messages by accounts with high bot scores are also retweeted by likely humans.

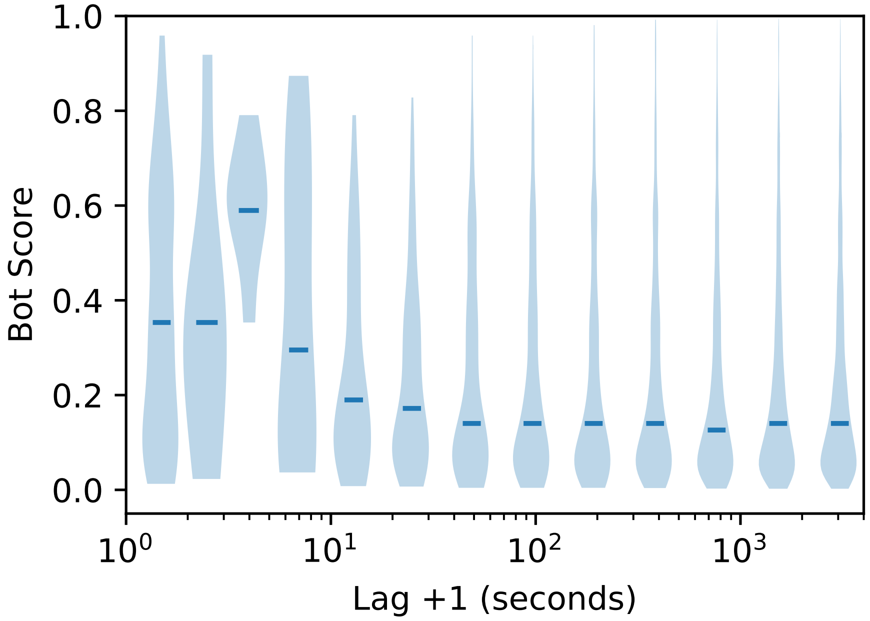

Supplementary Figure 11: Temporal evolution of bot support after the first share of a viral story from a consensus low-credibility source. We consider a random sample of 20,000 accounts out of the 163,563 accounts that participate in the spread of the 1,000 most viral articles. After articles from *The Onion* are excluded, we are left with 42,202 tweets from 13,926 accounts. We align the times when each link first appears. We focus on a one-hour early spreading phase following each of these events, and divide it into logarithmic lag intervals. The plot shows the bot score distribution for accounts sharing the links during each of these lag intervals.

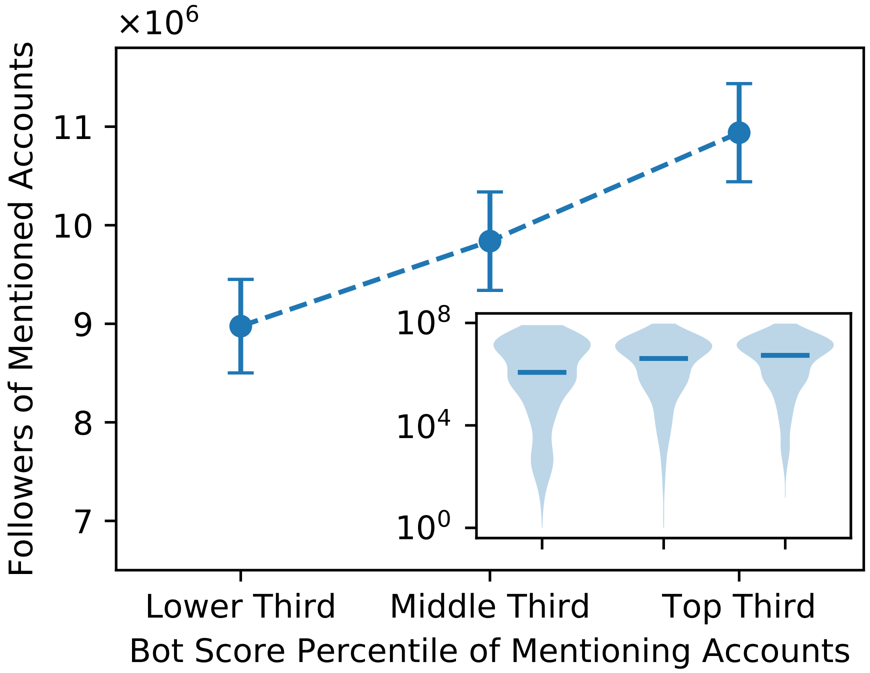

Supplementary Figure 12: Average number of followers for Twitter users who are mentioned (or replied to) by a sample of 20,000 accounts that link to the 1,000 most viral articles from consensus low-credibility sources. We obtained bot scores for 4,006 unique mentioning accounts and 4,965 unique mentioned accounts, participating in 33,112 mention/reply pairs. We excluded 13,817 of these pairs using the "via @screen_name" mentioning pattern. The mentioning accounts are aggregated into three groups by bot score percentile. Error bars indicate standard errors. Inset: Distributions of follower counts for users mentioned by accounts in each percentile group.

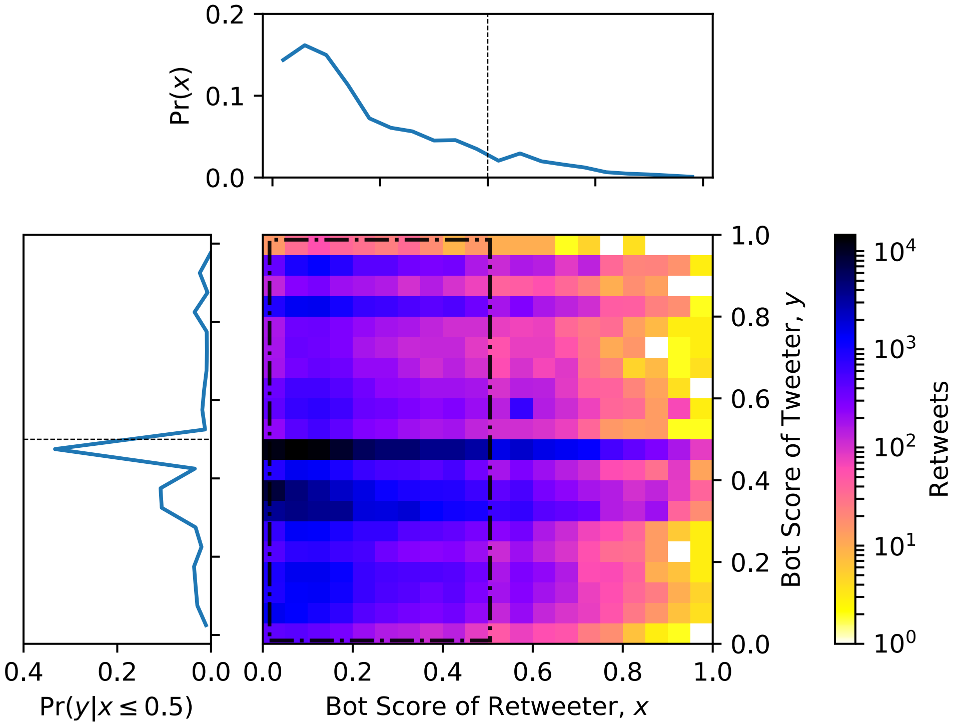

Supplementary Figure 13: Joint distribution of the bot scores of accounts that retweeted links to articles from consensus low-credibility sources and accounts that had originally posted the links. We considered retweets by a sample of 20,000 accounts that posted the 1,000 most viral articles. We obtained bot scores for 12,792 tweeting accounts and 17,664 retweeting accounts, participating in 229,725 retweet pairs. Color represents the number of retweeted messages in each bin, on a log scale. Projections show the distributions of bot scores for retweeters (top) and for accounts retweeted by likely humans (left).

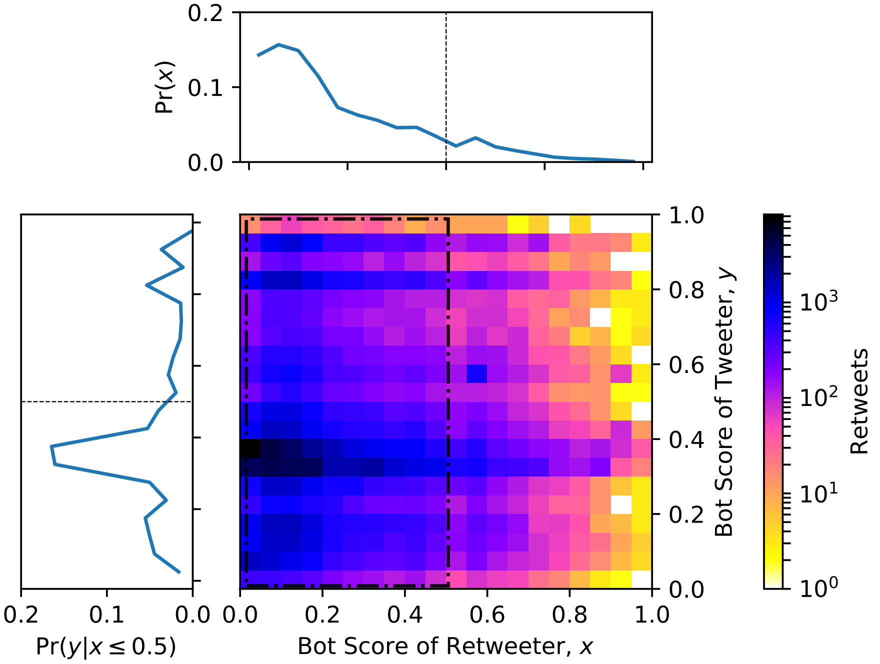

Supplementary Figure 14: Joint distribution of the bot scores of accounts that retweeted links to articles from consensus low-credibility sources and accounts that had originally posted the links. The analysis is identical to that in Supplementary Fig. 13, except that retweets of posts by a single account, `@PrisonPlanet`, are removed.

As shown in the main text, the volume of tweets with links to articles by different low-credibility sources is highly heterogeneous: a few sources are associated with millions of tweets each, representing a significant proportion of the overall volume of messages in the data we analyzed. To ensure that our findings are not driven by any one dominant source, we repeated the main analyses based on the consensus low-credibility sources, but excluding tweets with links to one of the most popular sites, namely `Breitbart.com`. The results, shown in Supplementary Fig. 15, are similar to those in the main text and those based on all consensus low-credibility sources (Supplementary Fig. 10–13), demonstrating our results are not dependent on a very popular source such as *Breitbart*.

Absence of correlation between activity and bot score

Our notion of super-spreader is based upon ranking accounts by activity and taking those above a threshold. The analysis about super-spreaders of low-credibility content being likely bots assumes that this finding is not explained by a correlation between activity and bot score. In fact, although the bot classification model does consider volume of tweets as one among over a thousand features, it is not trained in such a way that there is an obvious monotonic relation between activity and bot score. A simple monotonic relation between overall volume and bot score would lead to many false positives, because many bots produce very few tweets or appear to produce none (they delete their tweets); these accounts still get high bot scores. Supplementary Fig. 16 confirms that account activity volume and bot scores are uncorrelated.

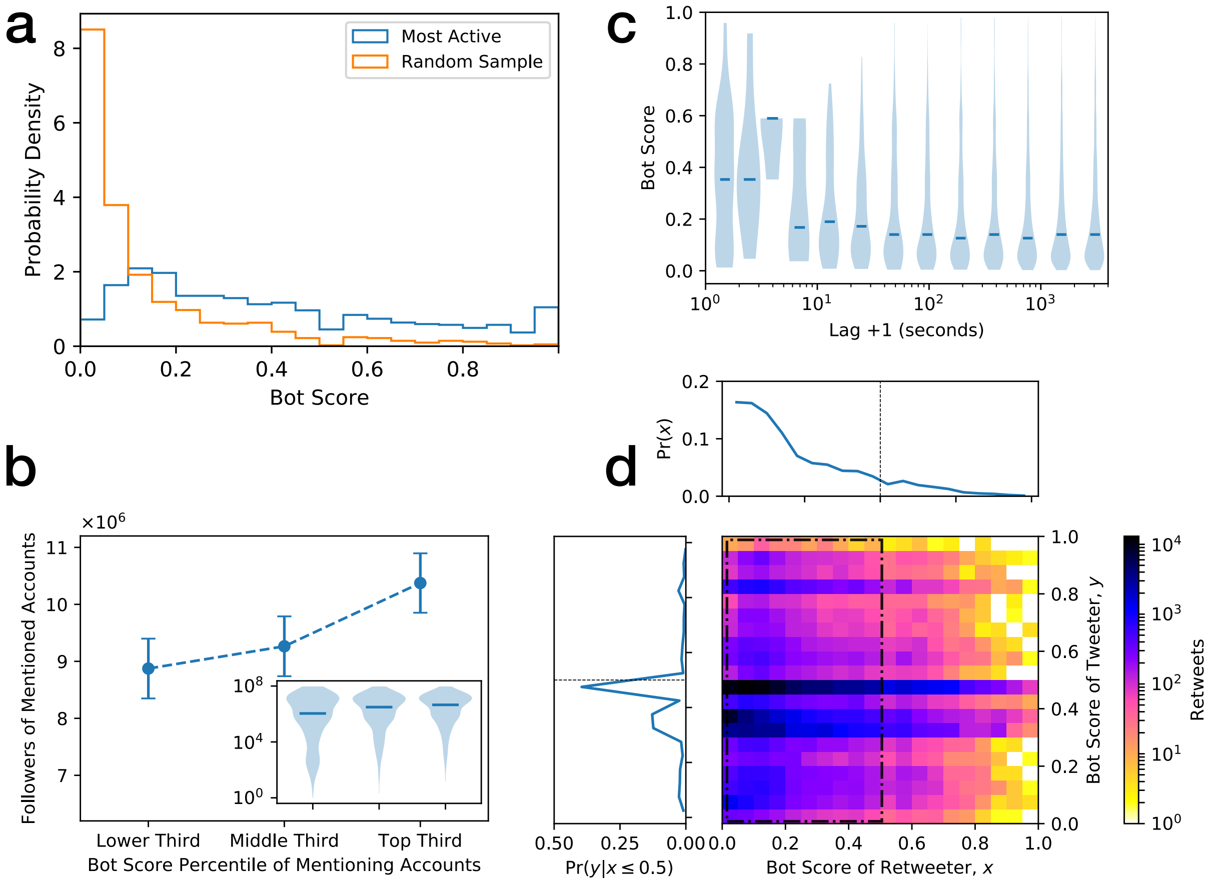

Supplementary Figure 15: Analyses based on consensus low-credibility sources with the exclusion of `Breitbart.com`. (a) Bot score distributions for super-spreaders vs. randomly selected sharers; see caption of Supplementary Fig. 10 for details. (b) Temporal evolution of bot support after the first share of a viral story; see caption of Supplementary Fig. 11 for details. (c) Average number of followers for Twitter users who are mentioned (or replied to) by accounts that link to viral low-credibility articles; see caption of Supplementary Fig. 12 for details. (d) Joint bot score distribution of retweeting and retweeted accounts; see caption of Supplementary Fig. 13 for details.

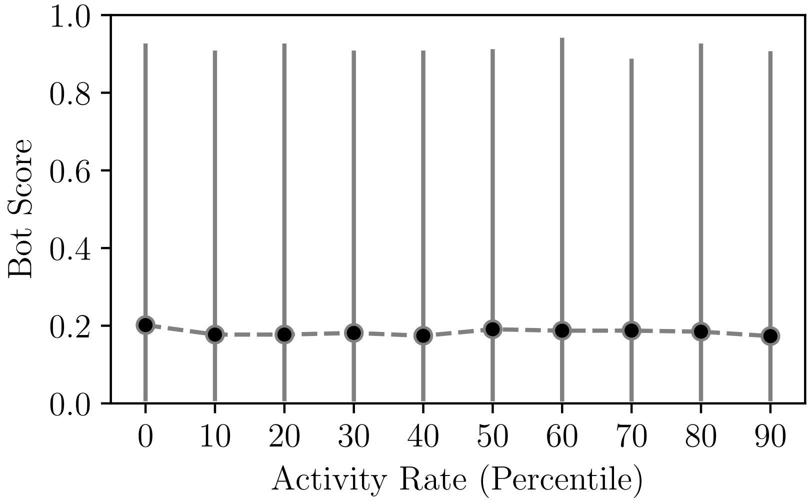

Supplementary Figure 16: Distributions of bot scores versus account activity. For this analysis we randomly selected 48,517 distinct Twitter accounts evaluated by Botometer. Of these, 11,190 were available for crawling their profiles and measuring their activity (number of tweets). Bins correspond to deciles in the activity rate. We show the average and 95% confidence interval for the bot score distribution of the accounts in each activity bin. There is no correlation between activity and bot score (Pearson's $\rho = -0.007$).

Bot-score threshold values

Finally, the results are not affected by the use of different bot-score thresholds to separate social bots and human accounts. For example, Supplementary Fig. 8 and 9 show that the findings about the amplification of low-credibility content by bots are robust with respect to the bot-score threshold, even though the estimated percentages of likely humans/bots, and the estimated numbers of tweets posted by them, are naturally sensitive to the threshold.

Background Tracking abuse of social media has been a topic of intense research in recent years. The analysis in the main text leverages Hoaxy, a system focused on tracking the spread of links to articles from low-credibility and fact-checking sources[4]. Here we give a brief overview of other systems designed to monitor the spread of misinformation on social media. This

is related to the problems of mining and detecting misinformation and fake news, which are the subjects of recent surveys[5,6].

Beginning with the detection of simple instances of political abuse like *astroturfing*[7], researchers noted the need for automated tools for monitoring social media streams and detecting manipulation or misinformation. Several such systems have been proposed, each with a particular focus or a different approach. The Truthy system[7] relied on network analysis techniques to classify memes, such as hashtags. TraceMiner[8] also models the propagation of messages, but by inferring embeddings of social media users with social network structures. The TweetCred system[9,10] focuses on content-based features and other kind of metadata, and distills a measure of overall information credibility. The Hierarchical Credibility Network[11] considers credibility as propagating through a three-layer network consisting of event, sub-events, and messages classified based on their features.

Specific systems have been proposed to detect rumors[12]. These include RumorLens[13], TwitterTrails[14], FactWatcher[15], and News Tracer[16]. The news verification capabilities of these systems range from completely automatic (TweetCred), to semi-automatic (TwitterTrails, RumorLens, News Tracer). In addition, some of them let the user explore the propagation of a rumor with an interactive dashboard (TwitterTrails, RumorLens). These systems vary in their capability to monitor the social media stream automatically, but in all cases the user is required to enter a seed rumor or keyword to operate them.

Our analysis is based on the spread of content from low-credibility sources rather than focus-

ing on individual stories that are labeled as misinformation. Due to the impossibility to fact-check millions of articles, this approach of using sources as proxies for misinformation labels is increasingly adopted in the literature cited in the main text, and more[1,17–20].

Since misinformation can be propagated by coordinated online campaigns, it is important to detect whether a meme is being artificially promoted. Machine learning has been applied successfully to the task of early discriminating between trending memes that are either organic or promoted by means of advertisement[21].

Finally, there is a growing body of research on social bot detection. The level of sophistication of bot-based manipulation can vary greatly[22]. As discussed in the main text, there is a large gray area between human and completely automated accounts. So-called cyborgs are accounts used to amplify content generated by humans[23]. It is possible that a significant portion of the manipulation discussed in this paper, aimed to amplify the spread of low-credibility content, is carried out by this kind of bot. The Botometer system used in this paper has been publicly available for a few years[24]. Its earliest version was trained on simple spam bots, detected through a social honeypot system[25,26]. The version used here was trained on public datasets that also included more sophisticated bots. A related problem is that of detecting camouflaged content polluters, accounts that maintain credibility by only devoting a small percentage of their posts to misinformation. Proposed defenses are based on detecting camouflage links[27,28] or camouflage content[29].

Supplementary References

1. Starbird, K. Examining the alternative media ecosystem through the production of alternative narratives of mass shooting events on twitter. In *Proceedings of the Eleventh International AAAI Conference on Web and Social Media (ICWSM)*, 230–239 (2017).

2. Niculescu-Mizil, A. & Caruana, R. Predicting good probabilities with supervised learning. In *Proceedings of the 22nd International Conference on Machine Learning*, 625–632 (2005).

3. DeGroot, M. H. & Fienberg, S. E. The comparison and evaluation of forecasters. *The statistician* 12–22 (1983).

4. Shao, C., Ciampaglia, G. L., Flammini, A. & Menczer, F. Hoaxy: A platform for tracking online misinformation. In *Proceedings of the 25th International Conference Companion on World Wide Web*, 745–750 (2016). URL `http://dx.doi.org/10.1145/2872518.2890098`.

5. Wu, L., Morstatter, F., Hu, X. & Liu, H. Mining misinformation in social media. In *Big Data in Complex and Social Networks*, 123–152 (CRC Press, 2016).

6. Shu, K., Sliva, A., Wang, S., Tang, J. & Liu, H. Fake news detection on social media: A data mining perspective. *ACM SIGKDD Explorations Newsletter* **19**, 22–36 (2017).

7. Ratkiewicz, J. *et al.* Truthy: Mapping the spread of astroturf in microblog streams. In *Proceedings of the 20th International Conference Companion on World Wide Web*, WWW '11, 249–252 (2011). URL `http://doi.acm.org/10.1145/1963192.1963301`.

8. Wu, L. & Liu, H. Tracing fake-news footprints: Characterizing social media messages by how they propagate. In *Proc. 11th ACM International Conference on Web Search and Data Mining (WSDM)* (2018).

9. Castillo, C., Mendoza, M. & Poblete, B. Information credibility on Twitter. In *Proceedings of the 20th International Conference on World Wide Web*, 675 (2011).

10. Gupta, A., Kumaraguru, P., Castillo, C. & Meier, P. Tweetcred: Real-time credibility assessment of content on twitter. In *International Conference on Social Informatics*, 228–243 (2014).

11. Jin, Z., Cao, J., Jiang, Y.-G. & Zhang, Y. News credibility evaluation on microblog with a hierarchical propagation model. In *Proc. IEEE International Conference on Data Mining (ICDM)*, 230–239 (2014).

12. Zubiaga, A., Aker, A., Bontcheva, K., Liakata, M. & Procter, R. Detection and resolution of rumours in social media: A survey. *ACM Computing Surveys* **50** (2018). Forthcoming.

13. Resnick, P., Carton, S., Park, S., Shen, Y. & Zeffer, N. Rumorlens: A system for analyzing the impact of rumors and corrections in social media. In *Proc. Computational Journalism Conference* (2014).

14. Metaxas, P. T., Finn, S. & Mustafaraj, E. Using twittertrails.com to investigate rumor propagation. In *Proceedings of the 18th ACM Conference Companion on Computer Supported Cooperative Work & Social Computing*, CSCW'15 Companion, 69–72 (2015).

15. Hassan, N. *et al.* Data in, fact out: Automated monitoring of facts by factwatcher. *Proc. VLDB Endow.* **7**, 1557–1560 (2014). URL http://dx.doi.org/10.14778/2733004.2733029.

16. Liu, X., Nourbakhsh, A., Li, Q., Fang, R. & Shah, S. Real-time rumor debunking on twitter. In *Proceedings of the 24th ACM International on Conference on Information and Knowledge Management*, CIKM '15, 1867–1870 (ACM, New York, NY, USA, 2015).

17. Vargo, C. J., Guo, L. & Amazeen, M. A. The agenda-setting power of fake news: A big data analysis of the online media landscape from 2014 to 2016. *New Media & Society* **20**, 2028–2049 (2018). URL https://doi.org/10.1177/1461444817712086.

18. Zannettou, S. *et al.* The web centipede: Understanding how web communities influence each other through the lens of mainstream and alternative news sources. In *Proceedings of the 2017 Internet Measurement Conference*, IMC '17, 405–417 (2017). URL http://doi.acm.org/10.1145/3131365.3131390.

19. Bessi, A. *et al.* Science vs conspiracy: Collective narratives in the age of misinformation. *PLoS ONE* **10**, 1–17 (2015). URL https://doi.org/10.1371/journal.pone.0118093.

20. Guess, A., Nyhan, B. & Reifler, J. Selective Exposure to Misinformation: Evidence from the consumption of fake news during the 2016 US presidential campaign. Unpublished manuscript (2018).

21. Varol, O., Ferrara, E., Menczer, F. & Flammini, A. Early detection of promoted campaigns on social media. *EPJ Data Science* **6**, 13 (2017). URL `https://doi.org/10.1140/epjds/s13688-017-0111-y`.

22. Boshmaf, Y., Muslukhov, I., Beznosov, K. & Ripeanu, M. The socialbot network: when bots socialize for fame and money. In *Proceedings of the 27th annual ACM computer security applications conference*, 93–102 (2011).

23. Chu, Z., Gianvecchio, S., Wang, H. & Jajodia, S. Who is tweeting on twitter: human, bot, or cyborg? In *Proceedings of the 26th annual ACM computer security applications conference*, 21–30 (2010).

24. Davis, C. A., Varol, O., Ferrara, E., Flammini, A. & Menczer, F. Botornot: A system to evaluate social bots. In *Proceedings of the 25th International Conference Companion on World Wide Web*, 273–274 (2016). URL `http://arxiv.org/abs/1602.00975`.

25. Webb, S., Caverlee, J. & Pu, C. Social honeypots: Making friends with a spammer near you. In *Proc. CEAS* (2008).

26. Lee, K., Caverlee, J. & Webb, S. Uncovering social spammers: social honeypots+ machine learning. In *Proceedings of the 33rd international ACM SIGIR conference on Research and development in information retrieval*, 435–442 (2010).

27. Hooi, B. *et al.* Fraudar: Bounding graph fraud in the face of camouflage. In *Proc. 22nd ACM International Conference on Knowledge Discovery and Data Mining (KDD)*, 895–904 (2016).

28. Wu, L., Hu, X., Morstatter, F. & Liu, H. Adaptive spammer detection with sparse group modeling. In *Proc. AAAI International CConference on Web and Social Media (ICWSM)*, 319–326 (2017).

29. Wu, L., Hu, X., Morstatter, F. & Liu, H. Detecting camouflaged content polluters. In *Proc. AAAI International CConference on Web and Social Media (ICWSM)*, 696–699 (2017).
